# Too sweet: cheminformatics for deglycosylation in natural products

**DOI:** 10.1186/s13321-020-00467-y

**Published:** 2020-11-04

**Authors:** Jonas Schaub, Achim Zielesny, Christoph Steinbeck, Maria Sorokina

**Affiliations:** 1 Institute for Inorganic and Analytical Chemistry, Friedrich-Schiller University, Lessing Strasse 8, 07743 Jena, Germany; 2 Institute for Bioinformatics and Chemoinformatics, Westphalian University of Applied Sciences, August-Schmidt-Ring 10, 45665 Recklinghausen, Germany

**Keywords:** Natural products, Sugars, Carbohydrates, Deglycosylation, Cheminformatics, Chemistry Development Kit, CDK

## Abstract

Sugar units in natural products are pharmacokinetically important but often redundant and therefore obstructing the study of the structure and function of the aglycon. Therefore, it is recommended to remove the sugars before a theoretical or experimental study of a molecule. Deglycogenases, enzymes that specialized in sugar removal from small molecules, are often used in laboratories to perform this task. However, there is no standardized computational procedure to perform this task in silico. In this work, we present a systematic approach for in silico removal of ring and linear sugars from molecular structures. Particular attention is given to molecules of biological origin and to their structural specificities. This approach is made available in two forms, through a free and open web application and as standalone open-source software.

## Introduction

Sugar is a general term that refers to a carbohydrate with the generic molecular formula C_n_(H_2_O)_n_. They are generally produced by living organisms and are mainly associated with sweet taste, but their function spreads way further than sweetening our palates. Indeed, only a few sugars, when in a solid or liquid state, taste “sweet”, among them glucose, fructose, or lactose. However, most of the sugar molecules are non-odorous and unsweet. Sugars are also very often found as substituents of small molecules produced by living organisms—for example, deoxyribose, the DNA building block. Furthermore, it is widely accepted [1–6] that sugar moieties are one of the most typical structural characteristics of biological molecules, and in particular of natural products (NPs). The latter are small molecules that have “higher” functions, such as signalling, intercellular and inter-organism communication, or defence. For instance, they inspire the pharmaceutical industry and research [7], and therefore their structural characteristics and the substituents from which they derive their activities are being intensely studied. There is evidence that the presence of stereo-diverse sugar units in aglycons (molecular structures without sugar substituents) affects their pharmacokinetic properties by making them more soluble [3] and being involved in transport, target specificity, in ligand-target interactions, and in particular, in the receptor binding [2, 8, 9]. However, in most of the cases, sugar units do not affect the principal activity of the aglycon [10] and importantly, may obstruct compound identification with experimental methods such as spectrometry. Therefore, when present or when the NP is not constituted only of them, sugar units are considered as redundant moieties and are often chemically or enzymatically removed from the parent structure [11, 12]. To optimize analyses of the aglycons, but also to computationally study their structures, one needs to predict their deglycosylated conformation and therefore remove the sugars informatically. Despite wide interest in the theoretical sugar removal from small biological molecules, there is currently no clear, complete, published, and widely accepted description on the process of such removal, neither online nor offline software that allows doing so in a convenient and fast way. Over the years, routines for the removal of furanoses and pyranoses linked by an O-glycosidic bond [13] and later, also by an N-glycosidic bond [14] have been described, but again, not made easily available to the public and were removing only very restricted types of sugar moieties.

Several challenges are to be faced in the task of theoretical deglycosylation: how exactly to define a sugar unit within a bigger structure, how to detect it, where exactly to cut the bond between the sugar unit and the parent structure, and how important and systematic are the glycosidic bonds between the parent structure and the sugar unit. An additional difficulty concerning any solution to these challenges is that they cannot be generally applicable to every case. Instead, their applicability depends on the specific analysis of the aglycon in the following step. This problem underlines that the question in theoretical deglycosylation is not whether a particular substructure is chemically a sugar but rather whether it is a redundant sugar-like structure that can obstruct the analysis of the parental structure within the molecule and can be removed without information loss. In several studies [13, 15, 16] and applications, such as Scaffold Hunter [17, 18], the “terminal ring sugars” are said to be removed, and this also demands a more thorough definition of what are terminal sugars and ring sugars. The latter suggests that there are also non-terminal and linear sugar substructures in molecular constructs.

**Fig. 1:**
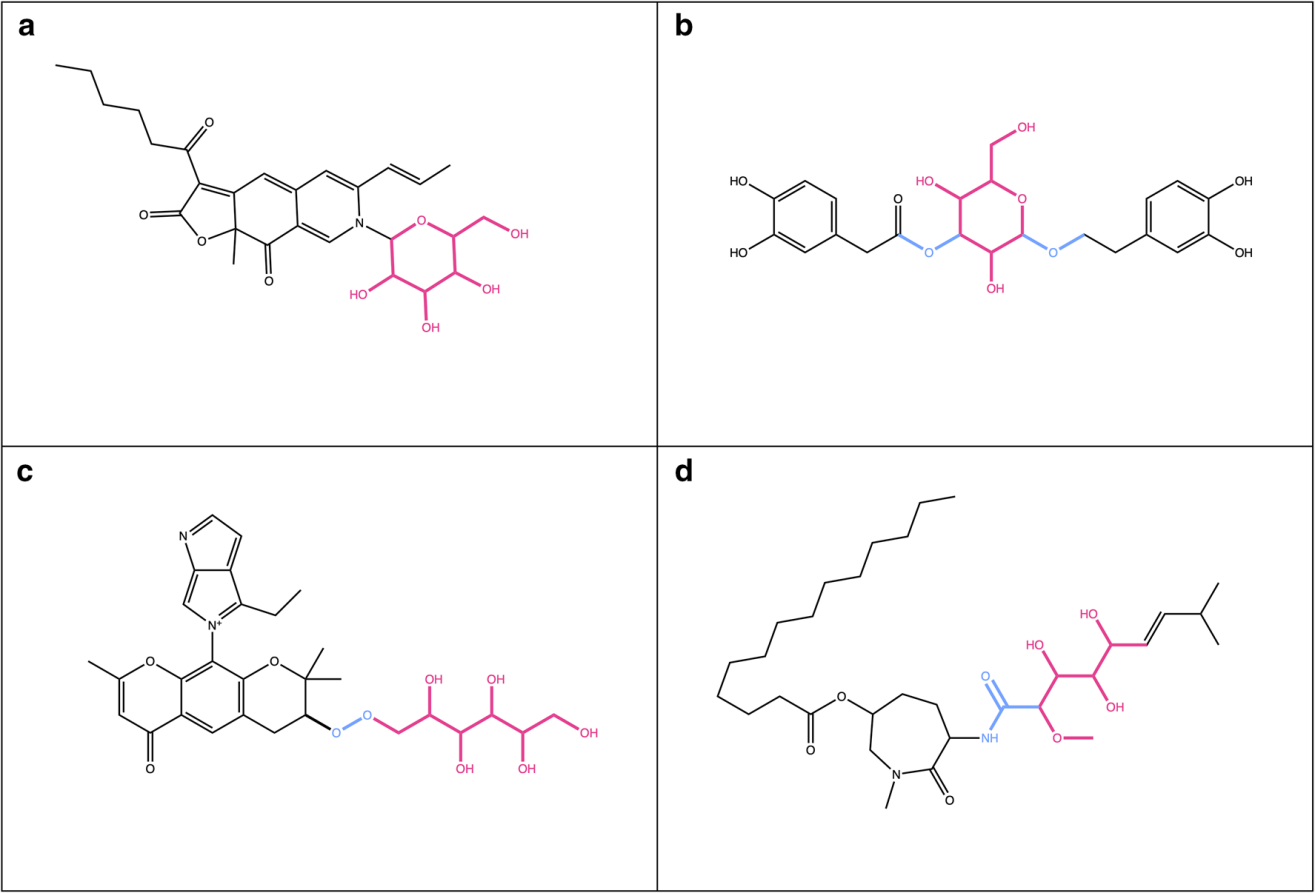
Examples of natural products with different types of sugars attached. **a** *N*-Glucosylrubropunctamine with a circular terminal sugar (in red). **b** Aquaticoside C, a natural product with a non-terminal circular sugar (in red) attached to the parent structure by O-glycosidic bonds (in blue). **c** 4-Ethyl-5-(2,2,8-trimethyl-6-oxo-3-((2,3,4,5,6-pentahydroxyhexyl)peroxy)-3,4-dihydro-2*H*,6*H*-pyrano[3,2-*g*]chromen-10-yl)pyrrolo[3,4-*b*]pyrrol-5-ium with a terminal linear sugar (in red) attached to the parental structure by a peroxide bond (in blue). **d** Bengamide B with a non-terminal linear sugar (in red) attached to the parental structure by an amide bond (in blue)

Here, we define a “terminal sugar” as a glycosidic substructure of a molecule, which when removed does not split the original molecule into two or more disconnected substructures (Fig. 1a, c). Opposed to it, a “non-terminal” sugar is a glycosidic substructure of a molecule, which can not be removed without disconnecting the remaining structure (Fig. 1b, d). Ring (circular) sugars are carbohydrate units of N carbons where N − 1 carbon atoms and one of the oxygens are forming a ring structure, and N is generally between four and eight (Fig. 1a, b in red). A typical example of a ring sugar is the ɑ-d-(+)-glucopyranose. A linear sugar is a carbohydrate chain of N carbons where only C1, Cn or side-chain oxygens form bonds with an atom that doesn’t belong to the atom set of the given unit, and n is between four and seven (Fig. 1c, d in red). One example of a linear sugar is the open-chain form of d-(+)-glucose.

In some, but not all, molecules that contain ring sugar moieties, the latter are attached by glycosidic bonds (Fig. 1b in blue). A glycosidic bond is typically formed between the hemiacetal or hemiketal group of a sugar unit and the hydroxy group of the aglycon. O-Glycosidic bonds are the most common in biological molecules, but C-, N- and S-glycosidic bonds are also frequently encountered. Linear sugars are often attached to the parent structure or to other sugars by a peroxide, ester or ether bond (Fig. 1c, d in blue).

In this manuscript, we present a generalized algorithm for automated sugar removal from molecular structures, implemented in an application called the Sugar Removal Utility (SRU). We discuss and implement the removal of ring and linear sugars, in both cases considering whether they are terminal or not, and if the former are linked to the parental structure by a glycosidic bond. The SRU is freely available and comes in two flavours: as a free and open web application, accessible at https://sugar.naturalproducts.net and as an open-source standalone command-line application downloadable at the web application homepage and on GitHub (https://github.com/JonasSchaub/SugarRemoval). The web application allows quick removal of sugars from submitted molecules with a balance between the offered options and pre-set, default, functions. The standalone command-line application allows the processing of an unlimited number of molecular structures and a larger variety of options that allow tuning “à la carte” the removal of glycosides depending on the user’s specific aims. The SRU was tested on the set of sugar moieties appearing in bacterial glycosylated natural products published by Elshahawi et al. [6] in order to evaluate its efficiency on a small manually curated dataset of glyco-moieties in NPs.

## Methods

### Algorithm

**Fig. 2:**
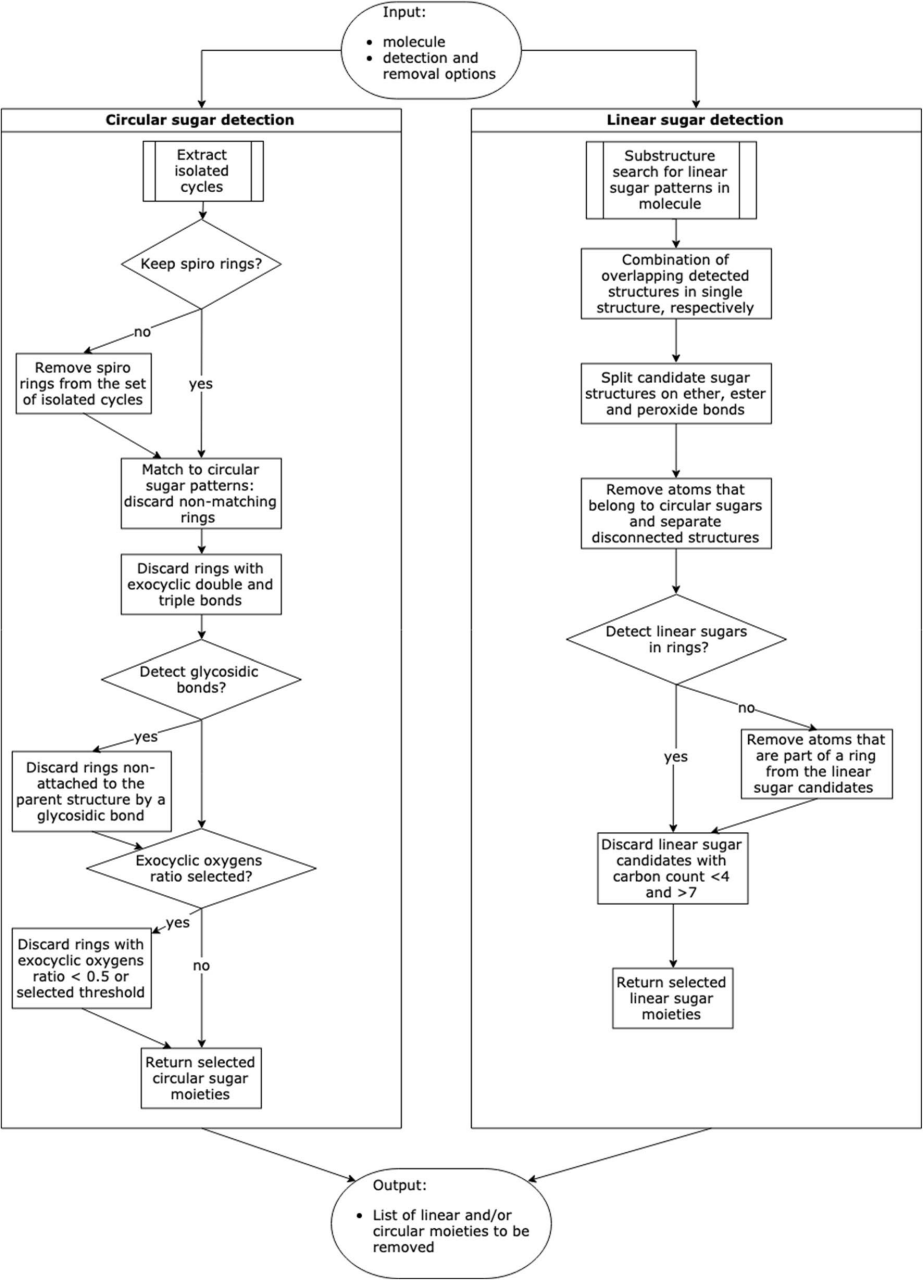
Workflow for circular and linear sugar detection in submitted molecules

**Fig. 3:**
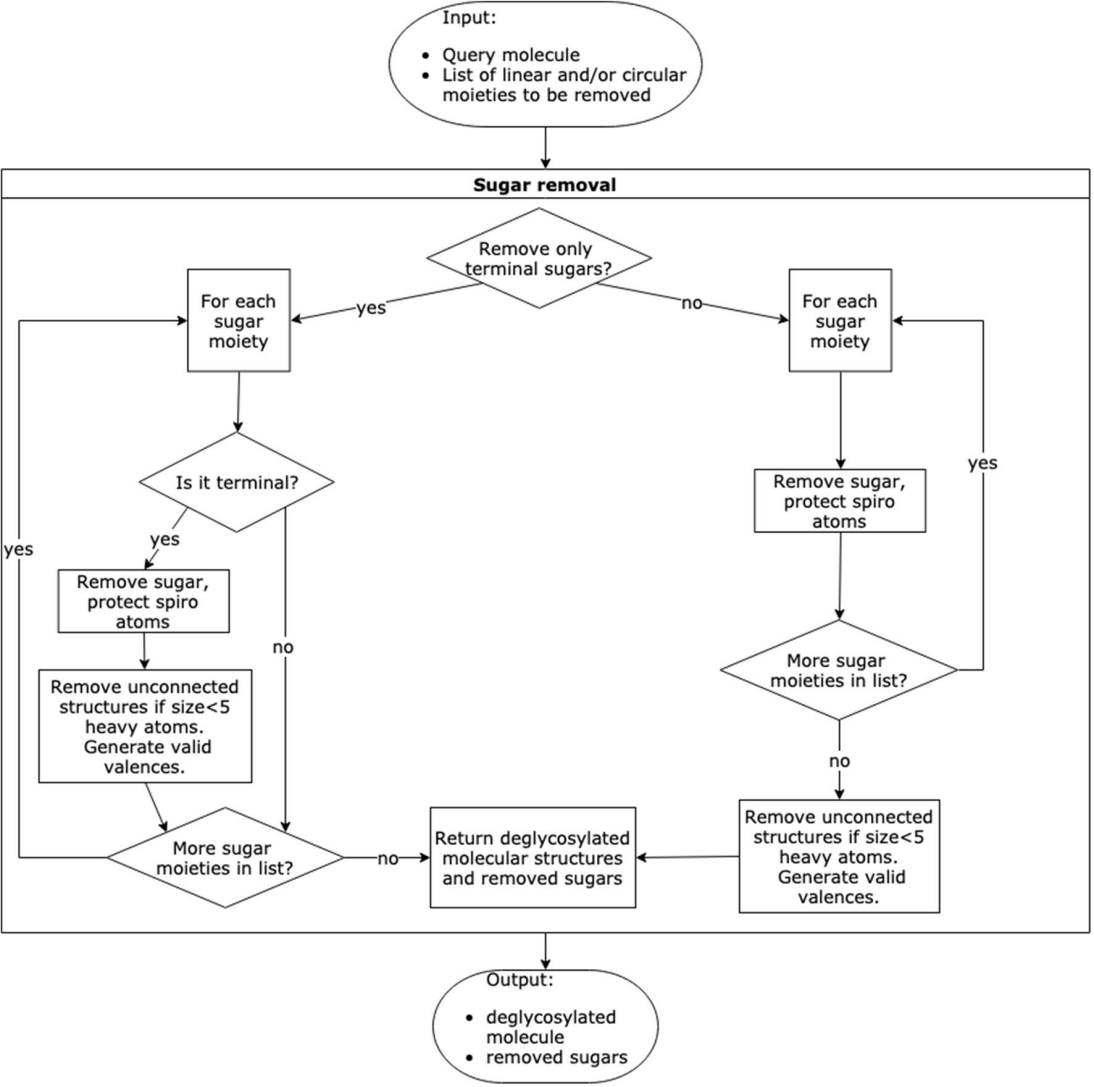
Workflow depicting the removal of a sugar moiety from a submitted molecule. This workflow depends on the output of the sugar detection workflow depicted in Fig. 2

The main implementation of the sugar removal algorithm is made in Java version 11 with the support of the Chemistry Development Kit (CDK) [19] version 2.3. It is downloadable on GitHub (https://github.com/JonasSchaub/SugarRemoval) along with the freely available code. The SRU offers multiple functionalities to detect and remove sugar moieties from submitted molecules along with a range of options to configure these processes for a specific application, summarised in Table 1. For greater modularity, the detection of sugar moieties (Fig. 2) and their removal (Fig. 3) are being done in different sequential steps, described below.

#### Detection of candidate structures for sugar moieties

The detection of glycosidic substructures in a query molecule is done distinctly for circular and linear sugar moieties in order to use specific approaches and detect these structurally different substructures in the most precise way possible.

##### Detection of circular sugar candidates

**Fig. 4:**
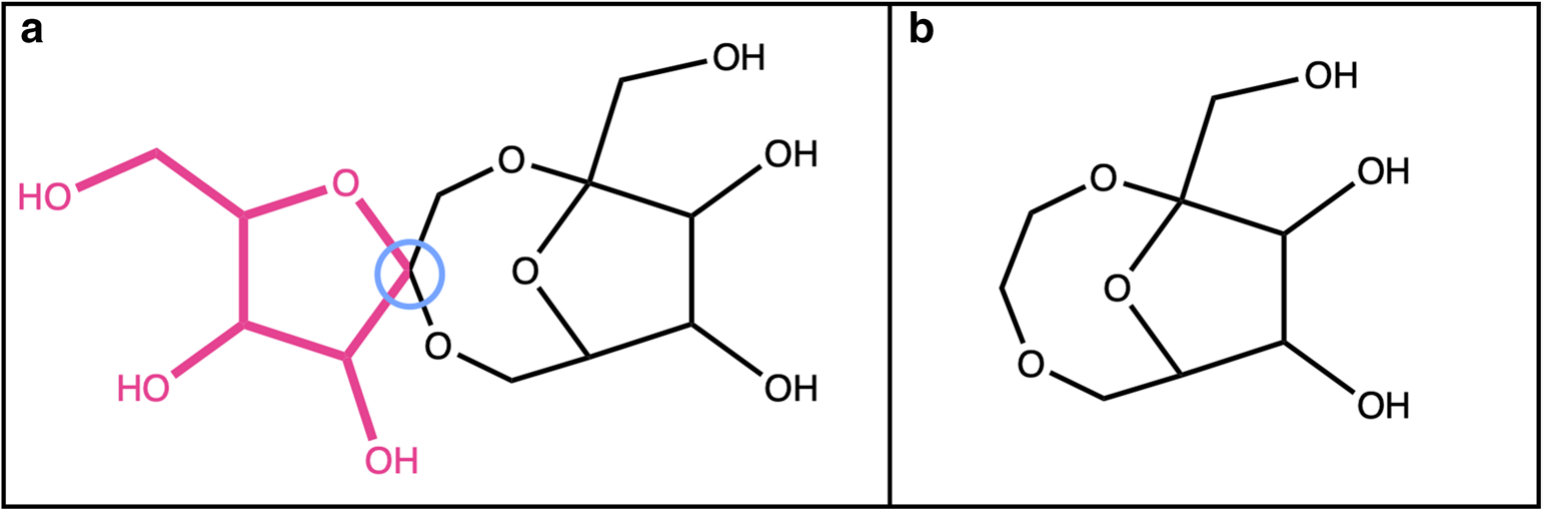
**a** 1,5′-bis(hydroxymethyl)dihydro-3′*H*-2,5,10-trioxaspiro[bicyclo[5.2.1]decane-4,2′-furan]-3′,4′,8,9-tetraol, in red a detected furanose attached to another cycle by a spiro carbon (attachment point inside the blue circle). **b** Remaining structure after removal of the sugar with preservation of the spiro carbon

**Fig. 5:**
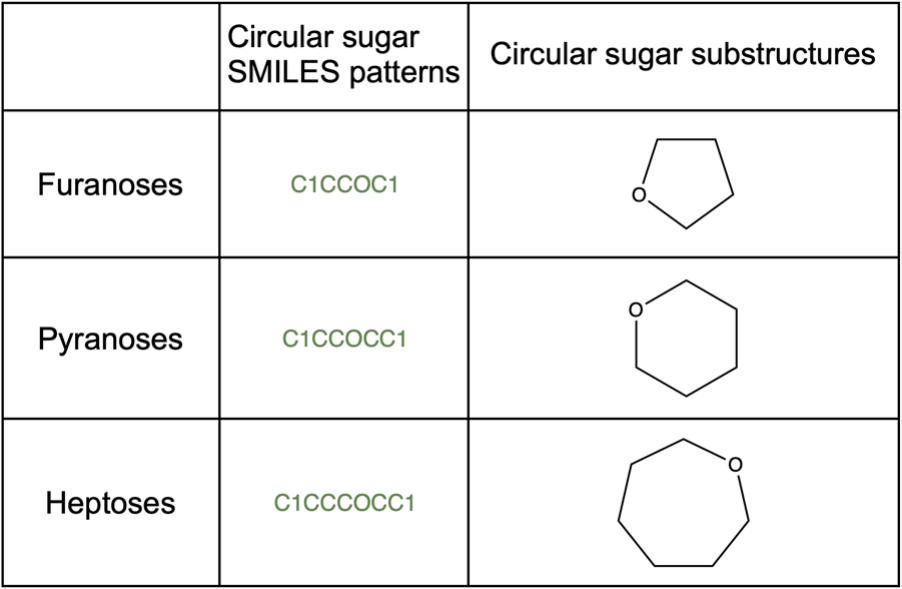
Circular sugar SMILES patterns and substructures

The detection of candidate structures for circular sugar moieties is done in three steps. First, using the CDK class *RingSearch*, all isolated cycles are extracted from the molecule. An isolated cycle has at most one atom in common with another cycle or cyclic system, as opposed to a ‘fused’ cycle that shares more atoms with others [20]. The definition of isolated cycles includes spiro ring systems where two cycles share one atom (Fig. 4a). These are filtered from the detected isolated rings but an option can be set to include them in the detection of circular sugars. Next, the detected cycles are matched to the predefined patterns for circular sugars. By default, these are tetrahydrofuran, tetrahydropyran, and oxepane matching five-, six-, and seven-membered sugar rings (Fig. 5). The SRU offers the option to add further rings to this list, like oxocane to match eight-membered sugar rings, or even to use one’s own collection of circular sugars to be detected. Only candidate moieties that match the given substructures are kept for the next step. Last, all rings that have exocyclic double or triple bonds are discarded.

Two additional options can also be selected for an even more specific circular sugar detection: counting of connected exocyclic oxygen atoms and detection of glycosidic bonds. If only sugars attached to the parental structure or to another sugar moiety by an O-glycosidic bond should be removed, this option should be selected. Sugar moieties having a carbon–carbon connection or an S-, C- N-glycosidic bond connecting them to other substructures in the molecule instead of an O-glycosidic bond are therefore preserved. Note, however, that molecules that are themselves single-cycle circular sugars are not discarded even with this option selected and still treated as sugar candidates to be removed because there is no other structure in the molecule to bind to via an O-glycosidic bond. It is also important to note that the algorithm detects glycosidic bonds as oxygen atoms connected to the sugar ring in any place and to another non-hydrogen atom via single bonds. This definition is not very strict and includes non-classical glycosidic linkages like ester bonds, for example (compare Fig. 1b in blue on the left-hand side of the circular sugar moiety).

The second optional circular sugar detection step consists in counting connected exocyclic oxygen atoms and discarding substructures that do not have a sufficient number of attached exocyclic oxygens. This sufficient number is defined by a ratio of connected exocyclic oxygen atoms to the number of atoms in the ring which can be configured in the SRU. A ratio of 0.5, for example, means that a six-membered suspected sugar ring needs at least three connected exocyclic oxygen atoms to be regarded as a sugar moiety that should be removed. All candidates not reaching this threshold are discarded and therefore not treated as removal-worthy sugar moieties. In the web application, the default threshold only is available.

All candidate structures for circular sugar moieties removal that have been selected in these steps are then being processed for sugar removal.

##### Detection of linear sugar candidates

**Fig. 6:**
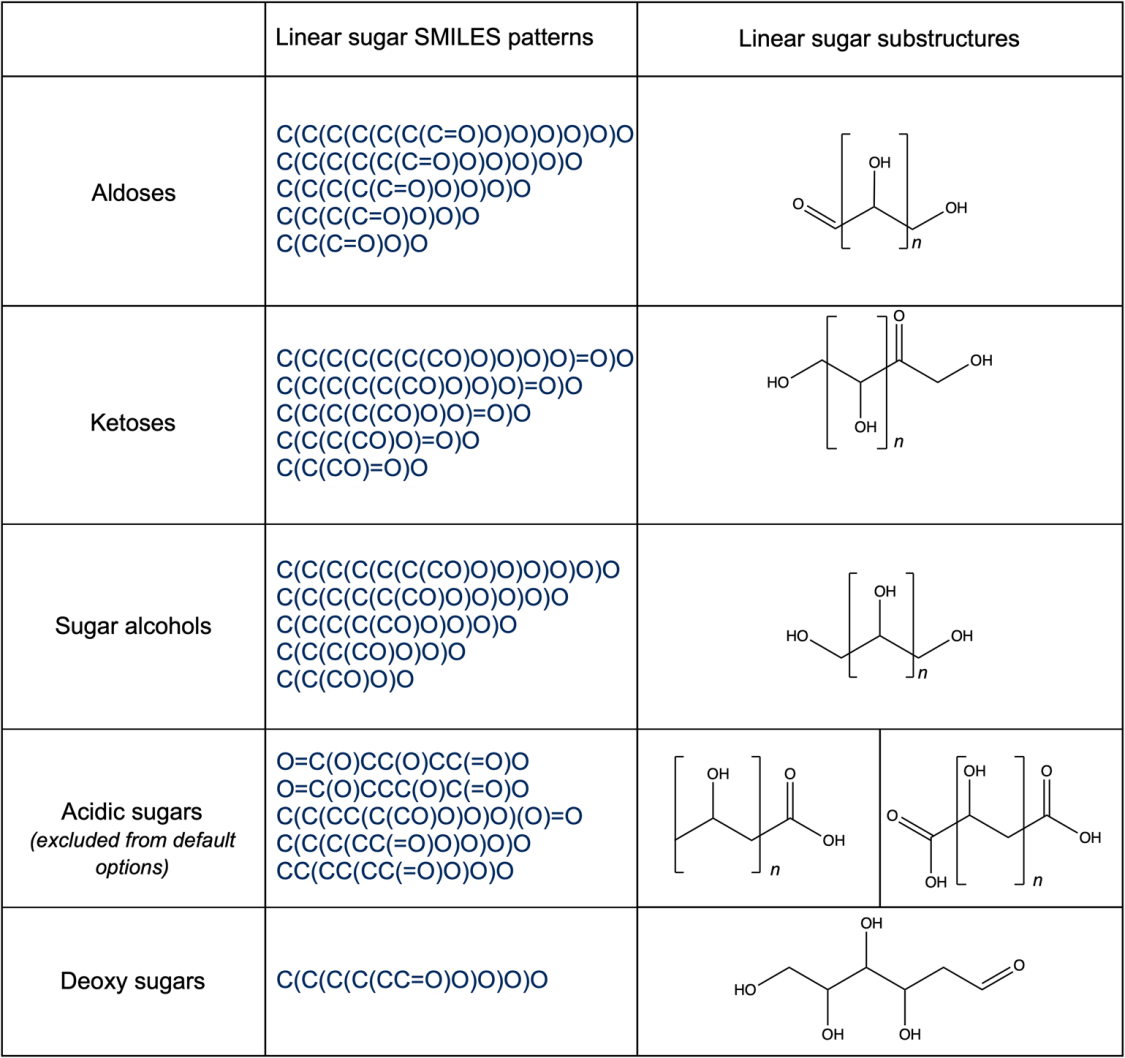
Linear sugar SMILES patterns and substructures

The detection of candidate structures for the presence of linear sugar moieties (single-bonded, simple carbon chains where nearly all carbon atoms have one hydroxy or keto group) is performed with a substructure matching against the whole molecule in five steps. First, a predefined set of linear sugar structures is matched to the query molecule using the CDK class *DfPattern* and all matching substructures are treated as primary linear sugar candidates. This predefined set contains multiple aldoses, ketoses, and sugar alcohols sized between 3 and 7 carbons (Fig. 6). It has been compiled with special regards to the occurrence of linear sugars in NPs and can be modified regarding specific needs. One possible modification of the set is the addition of five sugar-acid structures that are not included using the default options (Fig. 6).

**Fig. 7:**
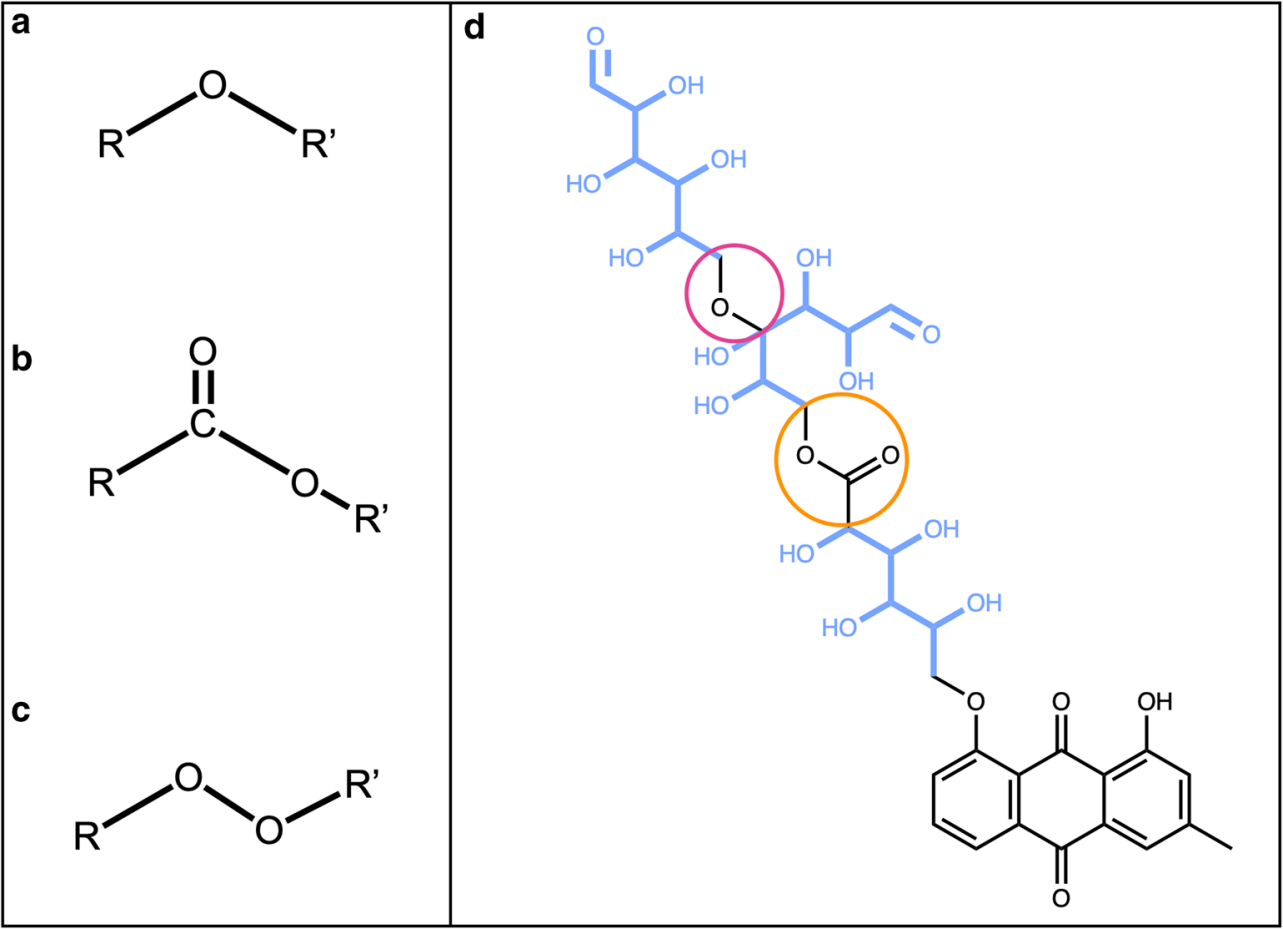
Linear sugars are mainly connected between them and to the parent structure with **a** an ether bond, **b** an ester bond, or **c** a peroxide bond. **d** 2,3,4,5-Tetrahydroxy-6-oxo-3-((2,3,4,5-tetrahydroxy-6-oxohexyl)oxy)hexyl 2,3,4,5-tetrahydroxy-6-((8-hydroxy-6-methyl-9,10-dioxo-9,10-dihydroanthracen-1-yl)oxy)hexanoate with the three linear sugars in blue, connected by an ether bond (red circle) and an ester bond (big orange circle)

**Fig. 8:**
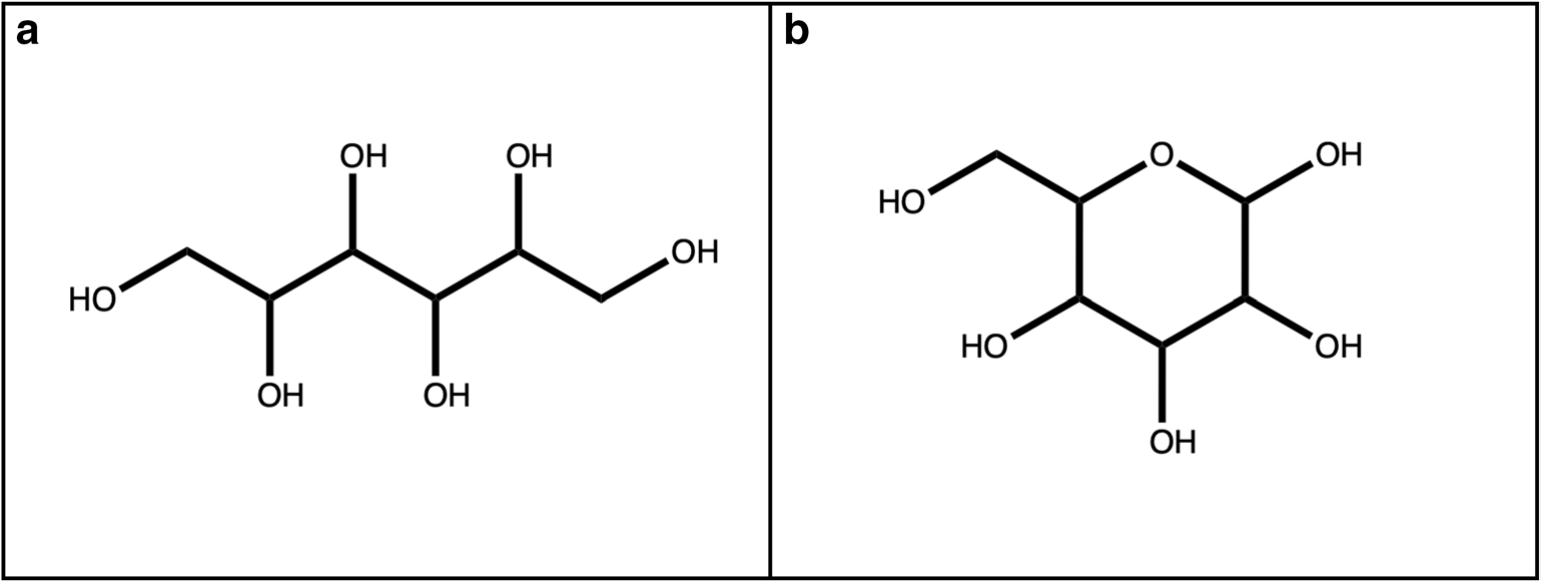
**a** Hexitol, six-membered sugar alcohol included in the linear sugar pattern set. **b** ɑ-d-Glucopyranose, six-membered ring sugar. Hexitol is a substructure of ɑ-d-glucose. Therefore, a sugar ring of this type gets extracted in an early stage of the linear sugar detection algorithm and needs to be rejected in the following steps

**Fig. 9:**
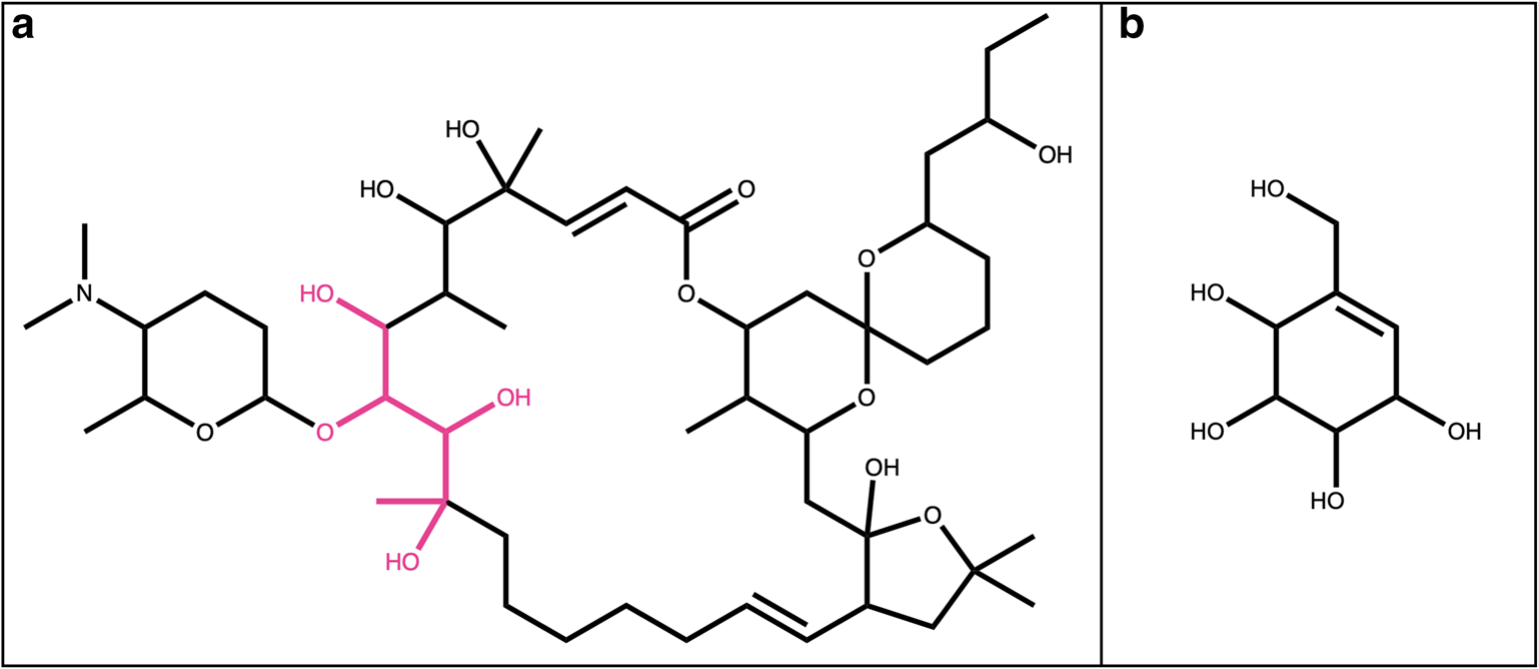
**a** Ossamycin. In red is marked the detected intra-macrocyclic linear sugar, whose removal will break the cycle. **b** Pseudosugar with a 6-membered ring, which structure can match with a linear sugar pattern

The substructures extracted by pattern matching in this first step may overlap, which can lead to ambiguities in the following steps. Therefore, in the second step, all overlapping candidates are combined to one single candidate structure. The output of this step is a set of distinct, non-overlapping sugar-like substructures of the query molecule. However, it may also combine substructures to one linear sugar candidate when they should be regarded as multiple, inter-linked sugar units. To separate these, in the third step candidates are split on ether, ester, and peroxide bonds (Fig. 7a–c) resulting into clean, distinct candidates (Figs. 1c, 7d). Only bonds that are located in a cycle are left intact to facilitate the detection of circular sugars among the linear sugar candidates in the following step. For example, the six-membered sugar alcohol hexitol (Fig. 8a), which is part of the linear sugar pattern set, matches an ɑ-glucopyranose sugar ring (Fig. 8b) and through the combination of overlapping matches, the whole sugar ring gets extracted as a linear sugar candidate. Therefore, to detect linear and circular sugar moieties separately, all atoms that are part of circular sugar moieties (i.e. isolated, non-spiro cycles that match the circular sugar patterns and have only exocyclic single bonds) are discarded. However, this does not guarantee that there cannot be any bigger cycles, or parts of them, in the remaining candidates for removal. For instance, in NPs, linear sugar moieties may be substructures of macrocycles, like it is the case in ossamycin (Fig. 9a). Pseudosugars (Fig. 9b), molecules that differ from true circular sugars only by the absence of an oxygen atom in their ring [6] are also to be cared of. They are undetectable for the presented circular sugar detection algorithm, but they can still be among the detected linear sugar candidates in this stage. The removal of these linear sugars would, therefore, break the macrocycles or pseudosugars. To avoid this, not removing linear sugars that are part of cycles is an optional step. When selected, all atoms in rings get removed from the candidate substructures for removal. Finally, the last step of the linear sugar detection is to check the length of the detected candidate substructures. By default, all linear sugars that have less than four and more than seven carbon atoms are discarded, but these thresholds can be manually configured in the standalone application. The algorithm returns all candidate structures for linear sugar moieties that have been selected as substructures that should be removed.

#### Removal of detected sugar moieties

**Fig. 10:**
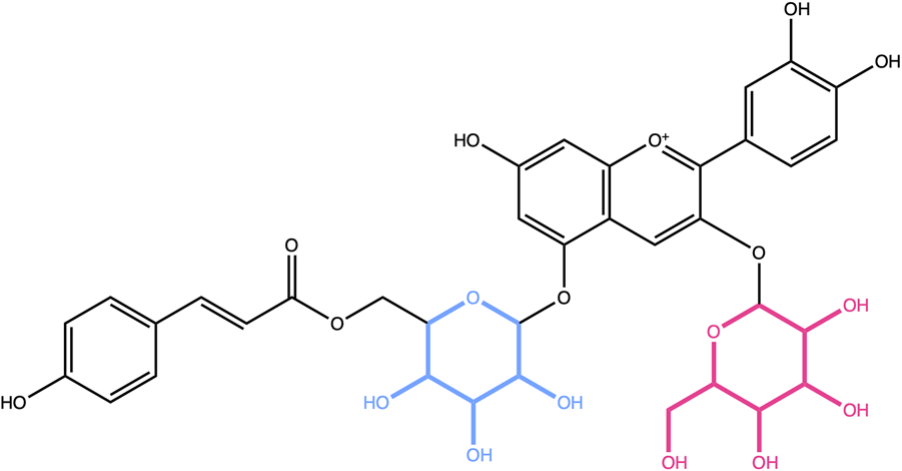
Cyanidin 3-galactoside-5-(6-*p*-coumarylglucoside) contains both terminal (red) and non-terminal (blue) pyranoses in its structure

**Fig. 11:**
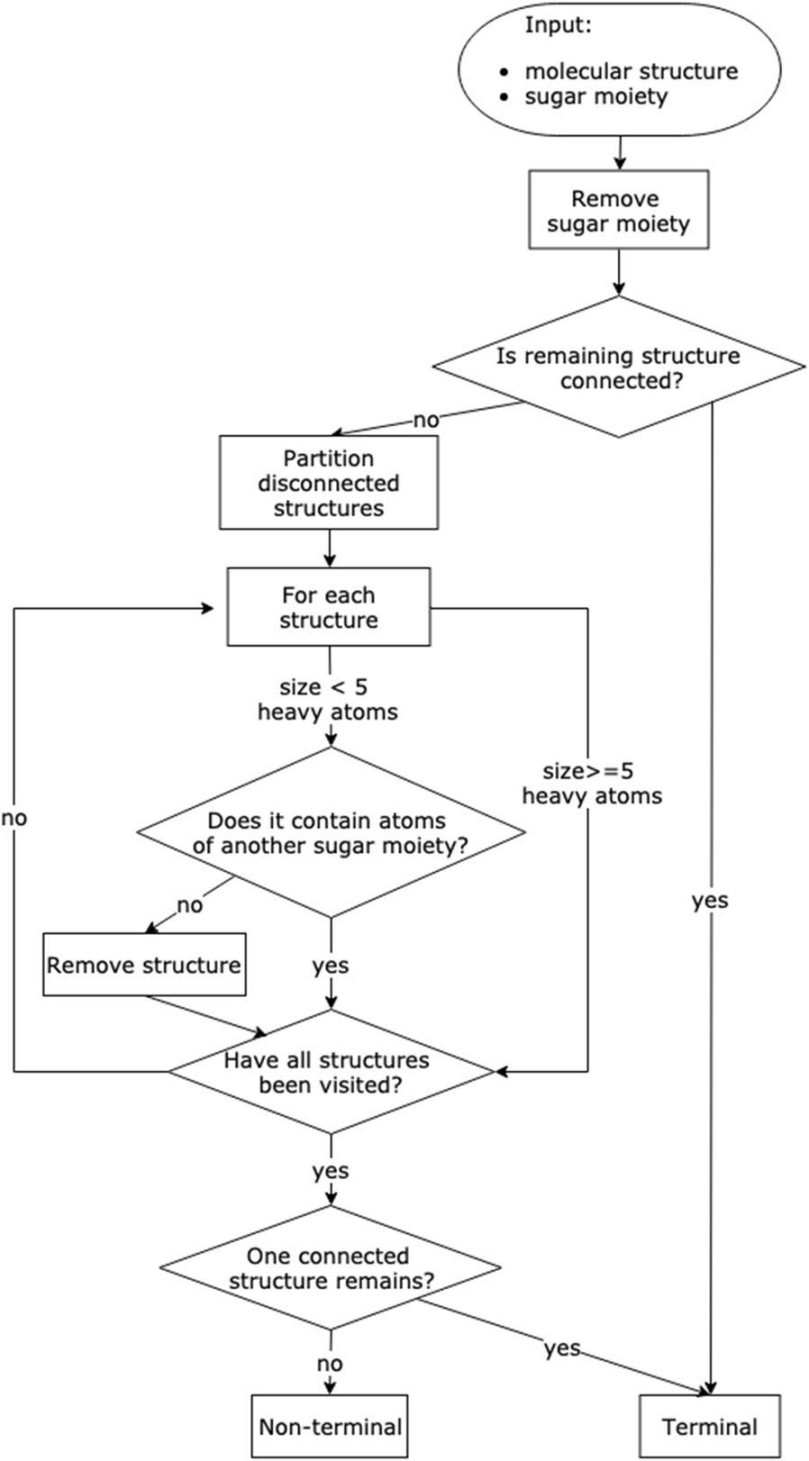
Workflow to determine if a sugar moiety is terminal or non-terminal

The removal of sugar moieties is comprised of the same steps for both linear and circular sugars. It is possible to remove all detected sugar moieties or only the terminal ones (Figs. 1a, b, 10, and 11). In the first case, the deglycosylated molecule may consist of two or more disconnected structures when returned. Whereas in the latter case, a recursive algorithm picks one candidate and removes it if it is terminal until no further terminal candidate can be found. The deglycosylated molecule is therefore always consisting of one connected structure.

The determination of terminal and non-terminal moieties heavily depends on an option named “preservation mode”. This option determines whether a substructure that gets disconnected from the molecule by the removal of a sugar moiety is worth keeping or can get removed along with the sugar. The best example where this is relevant is hydroxy groups of circular sugars. Following the algorithm presented above for the detection of circular sugars, these groups are not handled as part of the sugar candidate structure, even though their occurrence may be taken into account when deciding on whether to remove a sugar ring or not (see optional step above). When the ring is removed in this step, the hydroxy groups and all other structures formerly attached to the cycle get disconnected from the remaining structure. One-by-one, they are then evaluated according to the set preservation mode and removed or kept as disconnected structures. In the former case, the removed sugar ring qualifies as terminal, and in the latter case, it does not and therefore not get removed if only terminal sugar moieties are removed. For the determination of terminal sugar moieties, it is also a necessary condition that no structure belonging to another sugar candidate gets disconnected by the removal of the candidate in question.

The “preservation mode” has three different settings available.

1. Keep all structures. If only terminal moieties are removed, no sugar ring that has any hydroxy groups gets removed.
2. Judge by a heavy atom count threshold.
3. Judge by a molecular weight threshold.

These options are mutually exclusive and the default threshold values of the options 2 and 3 (five heavy atoms or 60 Da, respectively) can be altered.

If only terminal sugar moieties are to be removed from the molecule, any disconnected structure resulting from each removal step is too small to preserve according to the preservation mode and is cleared away. If all the candidate sugars are to be removed from the query molecule, the disconnected structures that are too small are only cleared once at the end of the routine. If multiple disconnected structures remain, routines of the SRU can be used either to select the biggest remaining substructure, or to split them in different entities and sort them. Note again, that when removing all circular and linear sugars, the routine is run only once; however, when removing only terminal sugars, the routine is iterated several times to ensure the unity of the parent structure. This is also done to detect and remove, for example, a linear sugar moiety that only becomes terminal after the removal of a circular moiety and vice-versa (Fig. 1a). This is the reason why the detection and removal of all terminal sugars at once may, in some particular cases, produce a slightly different deglycosylated parent structure compared to a sequential, detection and removal of circular, then linear sugars, which is also possible using the present implementation.

In the case where, for the detection of circular sugar moieties, the option was chosen to also detect spiro rings as possible sugar rings, the atom shared by one of these rings with another does not get removed in order to not break up the adjacent cycle (Fig. 4b).

**Fig. 12:**
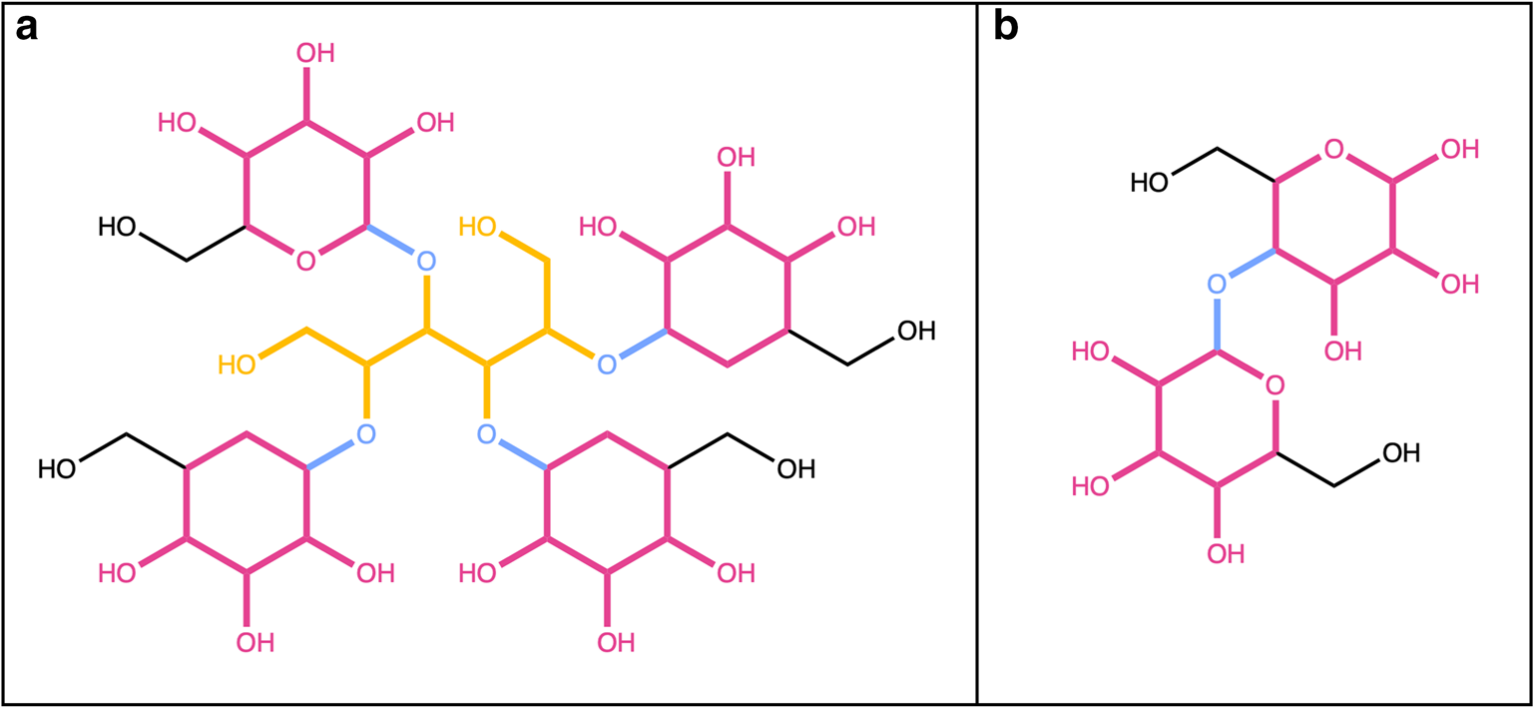
**a** 6-Hydroxy-2,3,4,5-tetrakis[[3,4,5-trihydroxy-6-(hydroxymethyl)oxan-2-yl]oxy]hexanal is composed of four pyranoses (in red) and one linear sugar (in yellow) connected by O-glycosidic bonds (in blue). **b** Lactose, composed of two pyranoses (in red) linked by a glycosidic bond (in blue)

A molecule only composed of sugars (Fig. 12) will be completely removed, and an empty object returned. However, if a molecule is composed of several sugar units that are not linked by O-glycosidic bonds, and the detection of O-glycosidic bonds is set, the query molecule will be returned unaltered. As mentioned before, only single-cycle carbohydrates must not adhere to this option set. In the case of commonly known sugars, like lactose (Fig. 12b) or sucrose, that are disaccharides linked by glycosidic bonds, both sugar moieties are detected and removed using the SRU.

Molecules that do not contain any of the sugar moieties selected for removal are returned unaltered.

#### Documentation availability

In Table 1 are summarised the multiple options that are available in the SRU, allowing to fine-tune the sugar detection and removal. Extensive documentation on the algorithm, different functions, options and option combinations is available on GitHub (https://github.com/JonasSchaub/SugarRemoval).

**Table 1:** Summary of available settings and options for fine-tuning the sugar detection and removal in the SRU, along with their impact, default, and availability

Setting	Options	Impact on	Default	Availability
Type of sugar moieties to remove	Circular/linear/both	Sugar removal	(None)	Web application, command-line application, source code
Remove only terminal sugars	Yes/no	Sugar removal	Yes	Web application, command-line application, source code
Preservation mode	Preserve all/judge by heavy atoms / judge by molecular weight	Sugar removal	Judge by heavy atoms	Command-line application, source code
Preservation mode threshold	Any number of heavy atoms or a molecular weight ≥ 0	Sugar removal	5 heavy atoms	Command-line application, source code
Detect circular sugars only with O-glycosidic bond	Yes/no	Circular sugar detection (and removal)	No	Web application, command-line application, source code
Detect circular sugars only with enough exocyclic oxygen atoms	Yes/no	Circular sugar detection (and removal)	Yes	Command-line application, source code
Exocyclic oxygen atoms to atoms in ring ratio threshold	Any ratio ≥ 0	Circular sugar detection (and removal)	0.5 (five- and six-membered sugar rings need at least 3 connected oxygen atoms)	Command-line application, source code
Detect spiro rings as circular sugars	Yes/no	Circular sugar detection (and removal)	No	Command-line application, source code
Detect linear sugars in rings	Yes/no	Linear sugar detection (and removal)	No	Command-line application, source code
Linear sugar candidate minimum size	Any number of carbon atoms ≥ 1	Linear sugar detection (and removal)	4 carbon atoms	Command-line application, source code
Linear sugar candidate maximum size	Any number of carbon atoms ≥ 1	Linear sugar detection (and removal)	7 carbon atoms	Command-line application, source code
Detect linear acidic sugars	Yes/no	Linear sugar detection (and removal)	No	Command-line application, source code
Circular/linear sugar patterns	Adding/removing patterns	Circular/linear sugar detection (and removal)	Pre-compiled set of circular and linear sugars (see Figs. 5 and 6)	Source code

### Web application

The single-page web application allowing to remove sugar units is freely available at https://sugar.naturalproducts.net/. It is implemented in Java 11 using Spring Boot MVC and JavaScript. The corresponding code for this web application is available at https://github.com/mSorok/SugarRemovalWeb. The web application implements all functionalities available in the standalone application, such as sugar removal of both linear and circular types and both terminal and non-terminal, with default options. For ring sugar removal, it is also possible to use the O-glycosidic bond option, to remove only sugars attached to the rest of the molecule by such a bond. The size of linear sugars to be removed is set between four and seven carbons and of ring sugars between five and seven atoms in the ring. Linear sugars that are part of bigger cyclic structures are not removed. Only deglycosylated substructures of more than 4 heavy atoms are returned. The query molecule submission is possible in three ways: by submitting a file (SDF, MOL or SMILES), by directly pasting a SMILES string, or by drawing the query molecular structure. The result of the deglycosylation is displayed in a table containing structures and SMILES representations of the submitted molecule(s) together with the produced deglycosylated moieties. The result table can be easily exported in a CSV format or copied to the clipboard.

## Results

### Detecting known bacterial sugars

In order to evaluate the capabilities of the Sugar Removal Utility to detect and consequently remove sugar moieties from NPs, a published, manually assembled dataset of over 344 mostly circular sugar moieties ([6] and Additional file 1) present in bacterial NPs was used. The aim was to check if the Sugar Removal Utility correctly detects all of the sugar moieties as such, a necessary step before their removal.

**Fig. 13:**
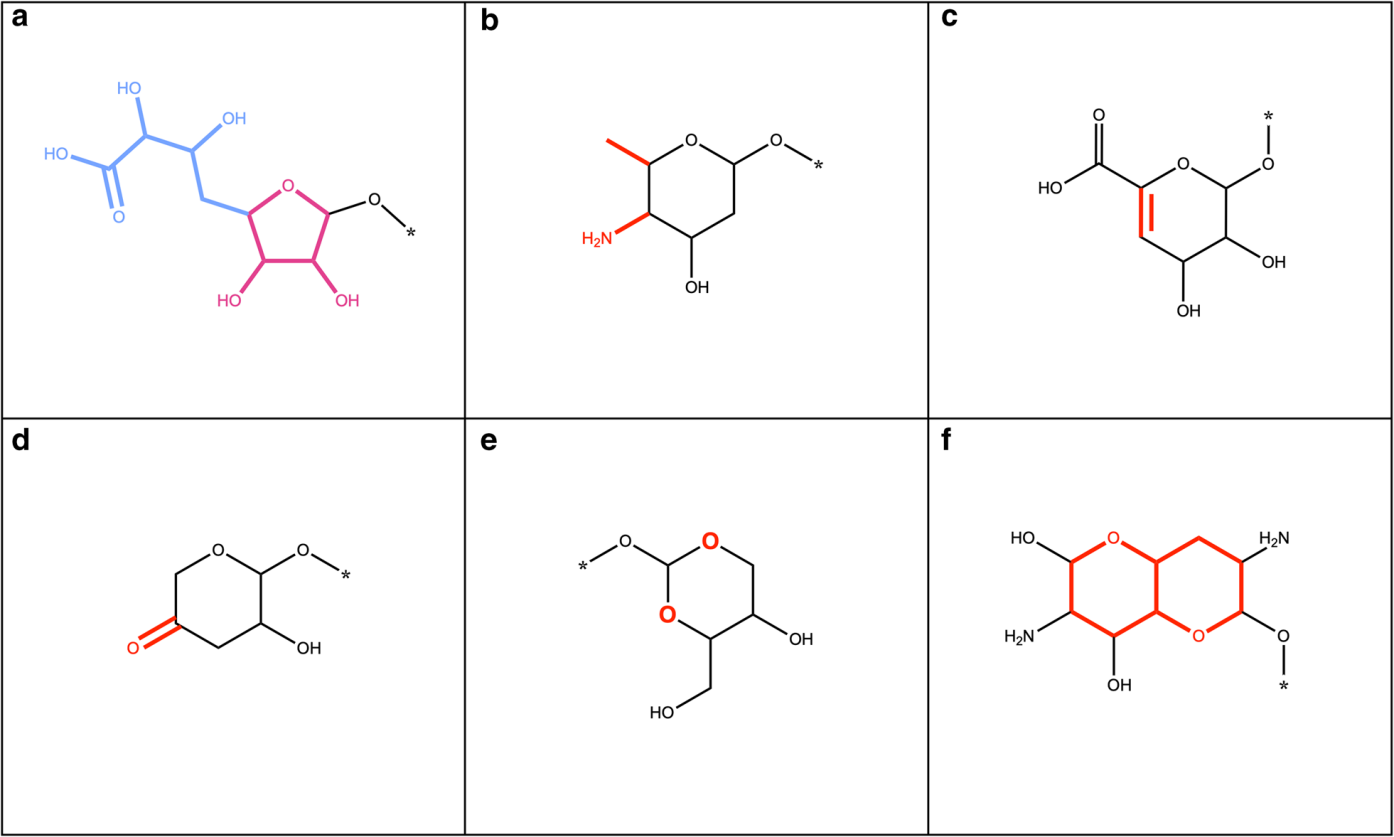
**a** 5′-Amino-5′-C-carboxy-5′-deoxy-β-d-ribofuranose contains a side chain of 5 heavy atoms (in blue) connected to the sugar ring (in pink). **b** 4′-Amino-2′,4′-dideoxy-β-l-fucose with only two oxygen atoms connected to the central sugar ring and extra nitrogen and methyl groups (in red). **c** 4′,5′-Unsaturated-α-d-mannuronic acid, not recognized as a sugar by the SRU because of the double bond in the sugar ring (in red). **d** 4′-Keto-3′,4′,5′-trideoxy-β-d-xylose with an exocyclic double bond in form of a keto group. **e** 4-(Hydroxymethyl)-1,3-dioxane-2,5-diol contains two oxygen atoms (in red) inside the ring. **f** 3,7-Diaminooctahydropyrano[3,2-b]pyran-2,4,6-triol is made up of a fused ring system (in red) that is not detectable by the SRU

With the default settings (i.a. remove circular and linear sugar moieties, neglect the absence of glycosidic bonds, remove only terminal sugars, detect only circular sugars with a sufficient amount of hydroxy groups, neglect linear sugars in rings), the SRU detected in this dataset 201 molecules that contain a sugar moiety, including one linear. 188 of them have been detected as pure sugar moieties, the 13 other entities contain either an additional side chain of more than 4 heavy atoms (Fig. 13a) or an isolated ring bigger than 4 heavy atoms in addition to the correctly detected and removed sugar moiety.

From the initial dataset, 143 entries were not recognized as sugar moieties by the SRU with the default settings. 118 do not have enough exocyclic oxygen atoms to be detected as a circular sugar moiety but decreasing the exocyclic oxygen ratio parameter in the SRU allows their detection. 4′-Amino-2′,4′-dideoxy-β-l-fucose in Fig. 13b, for example, and many other molecules in the data set can be detected as sugar moieties if the required minimum exocyclic oxygen ratio is set to 0.3 or less, instead of the default of 0.5. The reasons for the lack of detection of the other sugar moieties are the presence of a double bond in the ring (Fig. 13c), the presence of an exocyclic double bond (Fig. 13d), the presence of an additional heteroatom in the ring (oxygen or non-oxygen, Fig. 13e), and the presence of a non-isolated ring system (Fig. 13f). These molecular features do not correspond to the classical definition of glycosidic moieties and are considered as rare in many cases by the review authors themselves [6]. Since the central aim of the SRU is to remove redundant, sugar-like structures while preserving rare and characteristic structural features, the presented molecular traits are excluded from the definition of a sugar moiety in the SRU. Thus, the absence of detected sugar moieties is considered to be correct in these cases. Nevertheless, structures like those depicted in Fig. 13c, e can be detected by the SRU by adding corresponding structures to the set of predefined circular sugar patterns, an option accessible by using the SRU source code (see Table 1).

### Sugar removal in test cases

In addition to the detection of sugars known to be present in bacterial NPs, a small set of NPs with different types of sugar moieties has been hand-picked from public databases [21] in order to evaluate the performance and the different options of the SRU for particular sugar types and in special cases. The tests and the list of molecules itself are available at https://github.com/JonasSchaub/SugarRemoval (SugarRemovalUtilityTest.java).

**Fig. 14:**
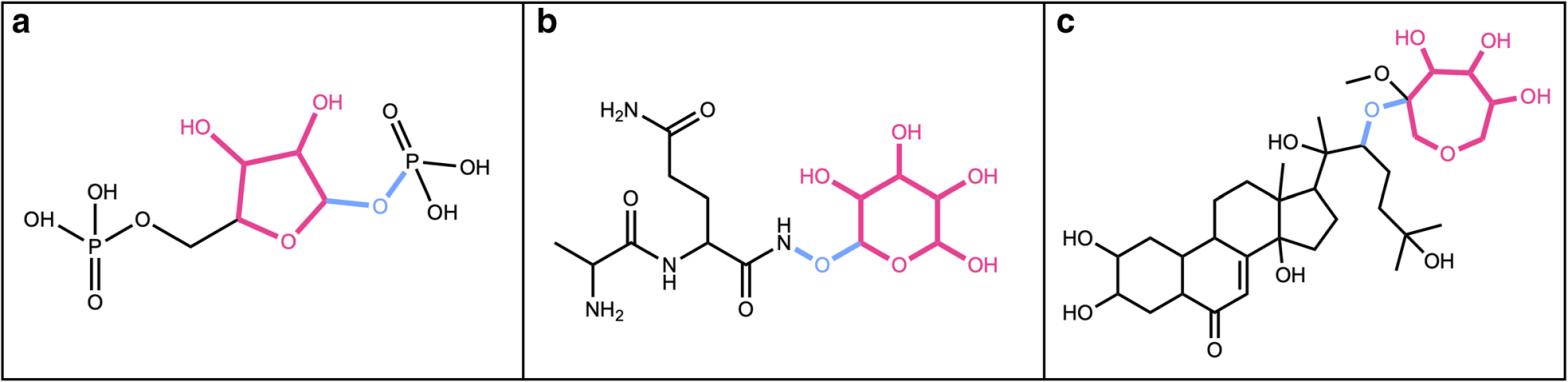
Detection of circular sugar moieties of different sizes. **a** 3,4-Dihydroxy-5-(phosphonooxy)tetrahydrofuran-2-yl)methyl dihydrogen phosphate with a 5-membered circular sugar (in red) and one glycosidic bond (in blue). **b** 2-(2-Aminopropanamido)-*N*^1^-((3,4,5,6-tetrahydroxytetrahydro-2*H*-pyran-2-yl)oxy)pentanediamide with a 6-membered circular sugar (in red) and a glycosidic bond (in blue). **c** 17-(2,6-Dihydroxy-6-methyl-3-((4,5,6-trihydroxy-3-methoxyoxepan-3-yl)oxy)heptan-2-yl)-2,3,14-trihydroxy-13-methyl-1,2,3,4,5,9,10,11,12,13,14,15,16,17-tetradecahydro-6*H*-cyclopenta[*a*]phenanthren-6-one with a 7-membered circular sugar (in red) and a glycosidic bond (in blue)

First, the detection and removal of ring sugars of the three predetermined sizes (5, 6 and 7 atoms) were successfully tested (Fig. 14). In the three cases, the glycosidic moiety was detected and removed.

**Fig. 15:**
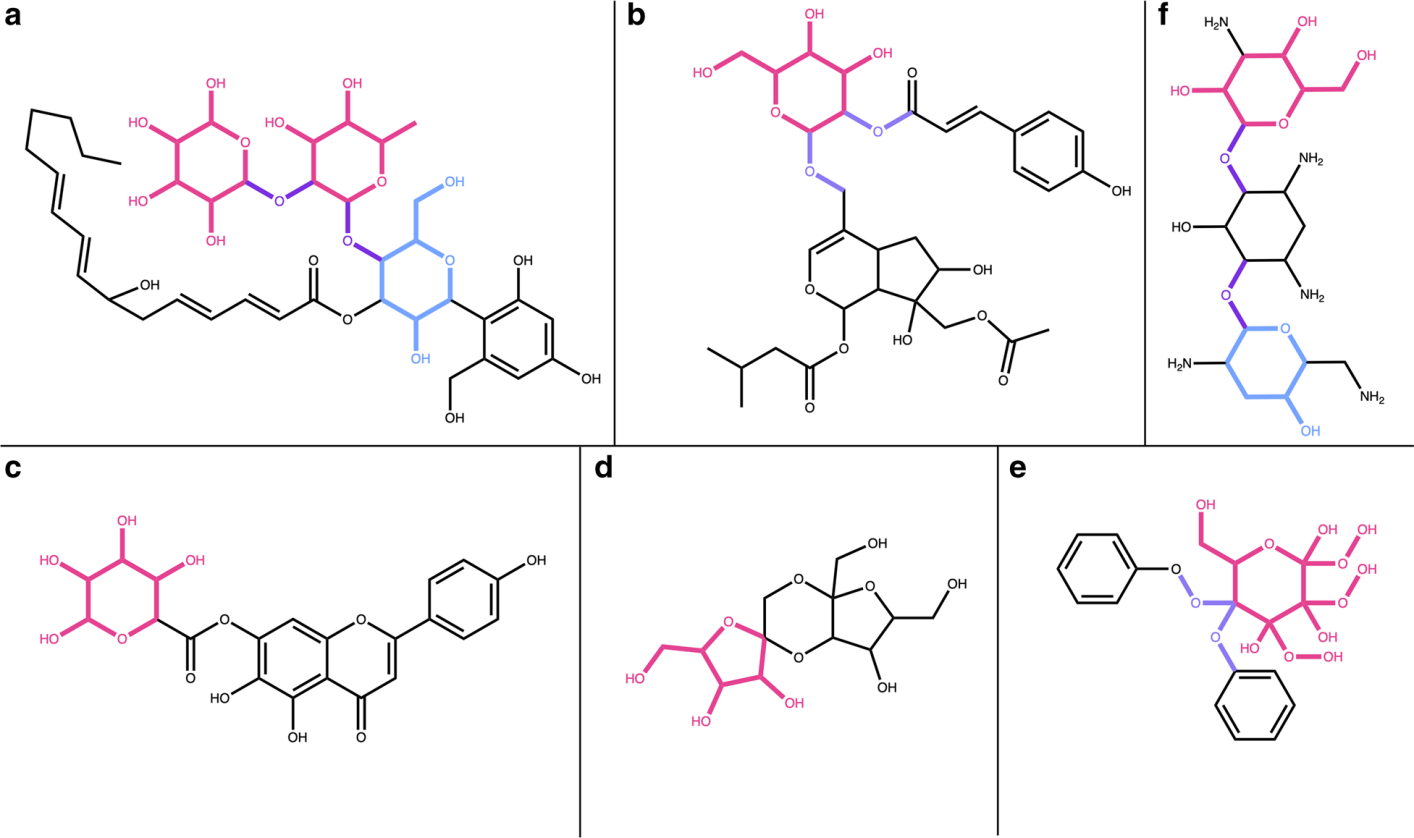
**a** Fusacandin B contains three circular sugars, two terminal (in red) and one non-terminal (in blue), all linked to the neighbouring structure by a glycosidic bond (in purple). **b** 7-[(Acetyloxy)methyl]-4-({[4,5-dihydroxy-6-(hydroxymethyl)-3-{[3-(4-hydroxyphenyl)prop-2-enoyl]oxy}oxan-2-yl]oxy}methyl)-6,7-dihydroxy-1*H*,4a*H*,5*H*,6*H*,7*H*,7a*H*-cyclopenta[c]pyran-1-yl 3-methylbutanoate with a non-terminal ring sugar (in red) linked by two glycosidic bonds (in purple) to the parental structures. **c** 5,6-Dihydroxy-2-(4-hydroxyphenyl)-4-oxo-4*H*-chromen-7-yl 3,4,5,6-tetrahydroxyoxane-2-carboxylate contains one terminal circular sugar (in red) that is not linked to the parent structure by a glycosidic bond. **d** 2,10-Bis(hydroxymethyl)-1,6,9,13-tetraoxadispiro[4.2.4^8^.2^5^]tetradecane-3,4,11,12-tetrol contains a circular sugar moiety (in red) that has a spiro carbon in its structure to link it to the rest of the molecule. **e** 2,3-Hexahydroxydiphenoxyl-glucose molecule with a non-terminal sugar ring (in red) where the ratio of exocyclic oxygens is bigger than 1 and that is linked to the parental structures by glycosidic bonds (in purple). **f** Tobramycin with two terminal ring sugars, linked by glycosidic bonds (in purple); the sugar in red has a ratio of exocyclic oxygens to atoms in the sugar ring ≥ 0.5 and the one in blue < 0.5

Next, we wanted to test the detection of terminal and non-terminal circular sugar moieties, the detection of glycosidic bonds, of spiro carbons in the cycle and of the ratio of exocyclic oxygens. Fusacandin B (Fig. 15a) contains 3 sugars: 2 terminal and one non-terminal, all attached to another sugar or to the parent structure by a glycosidic bond. When removing only terminal sugars, the third, non-terminal sugar (in blue) is not removed. It is however correctly removed when all sugars are to be detected and removed. The compound in Fig. 15b contains a non-terminal ring sugar attached by two glycosidic bonds to the parental structures. This sugar moiety is removed only when the options to remove all, including non-terminal, sugars is selected. The compound in Fig. 15c contains a six-membered terminal ring sugar that is not linked to the parent structure by a glycosidic bond. The sugar moiety is removed only when the option to detect glycosidic bonds is not selected, as it is in the default settings. The case of circular sugars linked to the parent structure with a spiro carbon is depicted in Fig. 15d: the 5-membered ring in red is not removed by default unless the detection of spiro rings is explicitly specified in the SRU parameters. And if it is removed, the spiro carbon is kept to avoid breaking the adjacent cycle. 2,3-Hexahydroxydiphenoxyl-glucose, shown in Fig. 15e is the perfect illustration of the case where the ratio of exocyclic oxygens to atoms of the sugar ring is higher than 1: with the default parameters it always gets detected and removed. On the opposite, tobramycin in Fig. 15f contains two circular sugars, but one (in blue) of them has a ratio of exocyclic oxygens to atoms in the sugar ring smaller than the default of 0.5 and therefore does not get removed with the default parameters.

**Fig. 16:**
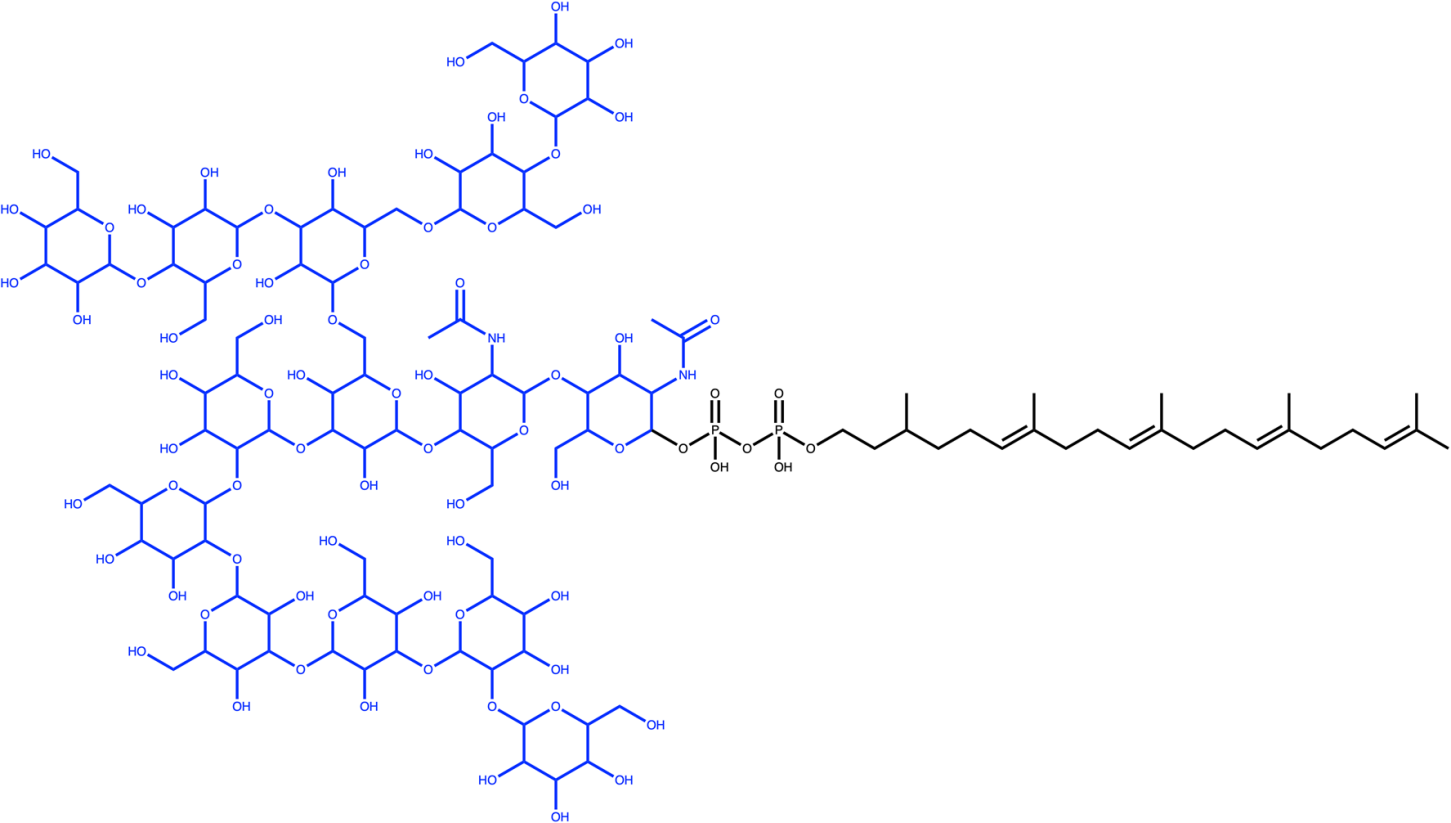
Glycan G00008, a dolichyl diphosphooligosaccharide composed of a branched tetradecasaccharide (in blue) attached to the dolichyl chain via a diphosphate linkage

To illustrate the SRU performance to detect numerous sugar moieties in a single molecule, glycan G00008 (Fig. 16), which contains 14 ring sugars attached to each other and to the parent structure by glycosidic bonds, was chosen. For the SRU, all the sugars in this molecule will be considered as terminal, as the sugar removal is recursive, therefore, all of them will be removed under the default parameters.

**Fig. 17:**
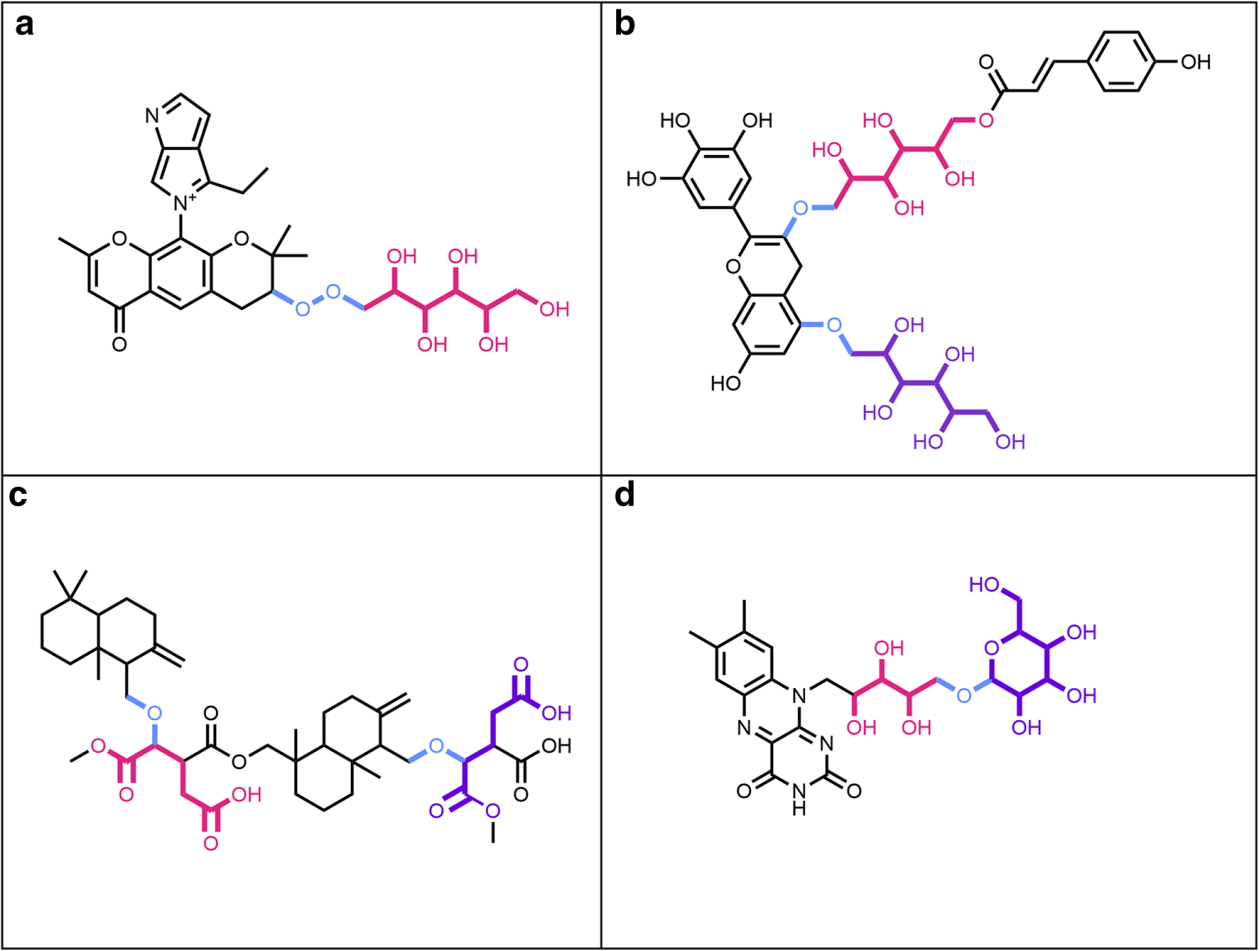
**a** 4-Ethyl-5-{2,2,8-trimethyl-6-oxo-3-[(2,3,4,5,6-pentahydroxyhexyl)peroxy]-2*H*,3*H*,4*H*,6*H*-pyrano[3,2-g]chromen-10-yl}-5λ^5^-pyrrolo[3,4-b]pyrrol-5-ylium contains a linear sugar aldose (in red) attached to the parental structure by a peroxide bond (in blue). **b** 2,3,4,5-Tetrahydroxy-6-({7-hydroxy-5-[(2,3,4,5,6-pentahydroxyhexyl)oxy]-2-(3,4,5-trihydroxyphenyl)-4*H*-chromen-3-yl}oxy)hexyl 3-(4-hydroxyphenyl)prop-2-enoate contains two aldoses, one terminal (in purple) and one non-terminal (in red) attached to the parent structure by ether bonds (in blue). **c** Cryptoporic acid F with two acidic sugars, one terminal (in purple) and one non-terminal (in red) attached to the parent structure by ether bonds (in blue). **d** 7,8-Dimethyl-10-(2,3,4-trihydroxy-5-{[3,4,5-trihydroxy-6-(hydroxymethyl)oxan-2-yl]oxy}pentyl)-2*H*,3*H*,4*H*,10*H*-benzo[g]pteridine-2,4-dione with an aldose (in red) and a ring sugar (in purple)

Then, the detection and removal of linear sugar moieties by the SRU were evaluated. First, the detection and removal of simple terminal linear sugars were successfully performed on the molecule from Fig. 7d. In this molecule, the three linear sugar moieties are attached between them and to the parent structure with ether and ester bonds and are considered as terminal, as their removal does not disrupt the parent structure. The molecule shown in Fig. 17a contains a simple terminal aldose attached to the parent structure with a peroxide bond; this glycoside will be detected and removed in all cases while searching for linear sugar moieties. The example shown in Fig. 17b demonstrates the case where a molecule also contains a non-terminal aldose: while searching to remove terminal sugars only, the purple sugar moiety will be removed, but not the red one. If all sugars are to be removed, both red and purple moieties will be detected and cleared from the parent structure. The cryptoporic acid F (Fig. 17c) contains two acidic sugars: these will not be removed under default parameters, as these have been purposely removed from the default linear sugar patterns. If included in these patterns, both moieties will be correctly removed according to the remaining options. The molecule shown in Fig. 17d was chosen to illustrate the conditional definition if a sugar is terminal or not. In this molecule there are two sugars, one circular (in purple) and one linear (in red); the circular sugar will be detected as terminal in all cases. However, the linear sugar will be detected as terminal only if all sugars are to be removed: if the user searches to detect and remove only terminal linear sugars, the aldose here will not be removed.

**Fig. 18.**
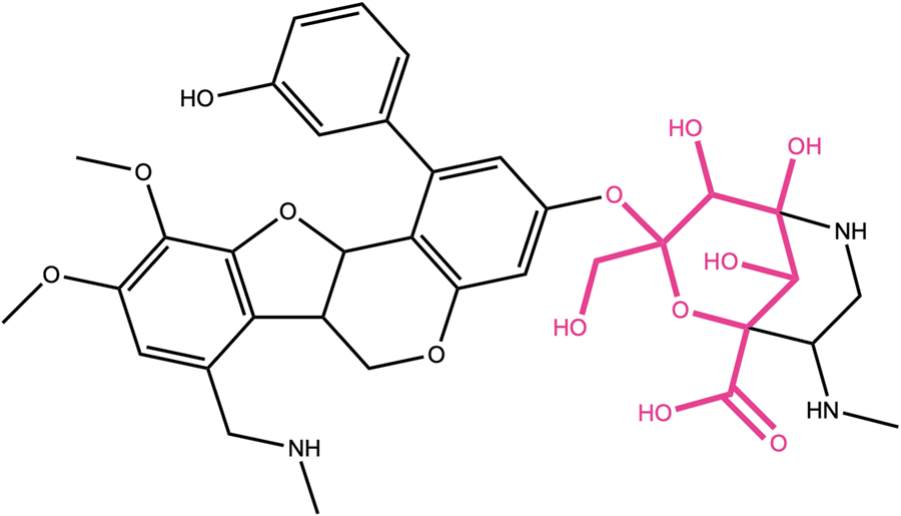
4,5,9-Trihydroxy-3-(hydroxymethyl)-3-{[3-(3-hydroxyphenyl)-14,15-dimethoxy-12-[(methylamino)methyl]-8,17-dioxatetracyclo[8.7.0.0^2^,^7^.0^11^,^16^]heptadeca-2(7),3,5,11(16),12,14-hexaen-5-yl]oxy}-8-(methylamino)-2-oxa-6-azabicyclo[3.3.1]nonane-1-carboxylic acid contains a macrocycle in which linear sugar patterns matched (in red)

While testing the SRU for the detection of linear sugars, the latter were often found as part of macrocycles (e.g. in macrolides). In order to avoid breaking these biologically and chemically relevant molecular structures, the possibility of detecting and removing linear sugar moieties that are part of cycles has been added as an option and by default, these sugars are not removed. The molecule shown in Fig. 18 matched with linear sugar patterns: it is however clear that the removal of this glycoside will break the macrocycle. Therefore, it is not recommended to switch on the option of detecting and removing linear sugars in cycles, unless the user is, for example, more interested in reducing the hydroxy content of the molecules and cares less about the defining structural features of the aglycon.

**Fig. 19:**
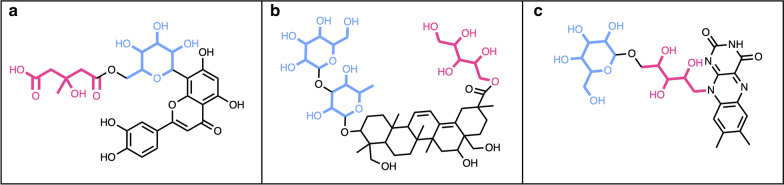
Natural products with both linear (in red) and circular (in blue) sugars in their structure. **a** 5-({6-[2-(3,4-Dihydroxyphenyl)-5,7-dihydroxy-4-oxo-4*H*-chromen-8-yl]-3,4,5-trihydroxyoxan-2-yl}methoxy)-3-hydroxy-3-methyl-5-oxopentanoate contains an acidic sugar (in red) and a simple circular sugar (in blue). **b** 5-*O*-[(3β,5ξ,9ξ,16α)-3-{[6-Desoxy-3-*O*-(β-d-glucopyranosyl)-β-d-galactopyranosyl]oxy}-16,23,28-trihydroxy-29-oxooleana-11,13(18)-dien-29-yl]-d-ribitol with two terminal ring sugars (in blue) connected between them and to the aglycon with glycosidic bonds and a terminal linear sugar alcohol. **c** 7,8-Dimethyl-10-(2,3,4-trihydroxy-5-((3,4,5-trihydroxy-6-(hydroxymethyl)tetrahydro-2*H*-pyran-2-yl)oxy)pentyl)benzo[g]pteridine-2,4(3*H*,10*H*)-dione contains a terminal circular sugar (in blue) and a non-terminal sugar alcohol (in red)

Natural products sometimes have both linear and circular sugar moieties in their structures, therefore, the SRU has also been tested on molecules presenting such structural features. In Fig. 19 are shown the different possible cases, where circular and linear sugar moieties have different terminal status, and their respective successful removal depends on the selected detection and removal options. For example, the molecule in Fig. 19a has a ring sugar that is considered as non-terminal if the search is only done for ring sugars; the acidic linear sugar in its structure is terminal but will be removed only if this type of sugars is added to the detected pattern list. In the molecule shown in Fig. 19b, all circular and linear sugars are terminal and will be removed while detecting the respective sugar types. Finally, the molecule in Fig. 19c contains one terminal ring sugar and a linear sugar alcohol, that will be detected and removed either when all terminal sugars are to be removed or when all linear sugars are to be removed. If only removing linear terminal sugars, this moiety will not be deleted.

**Fig. 20:**
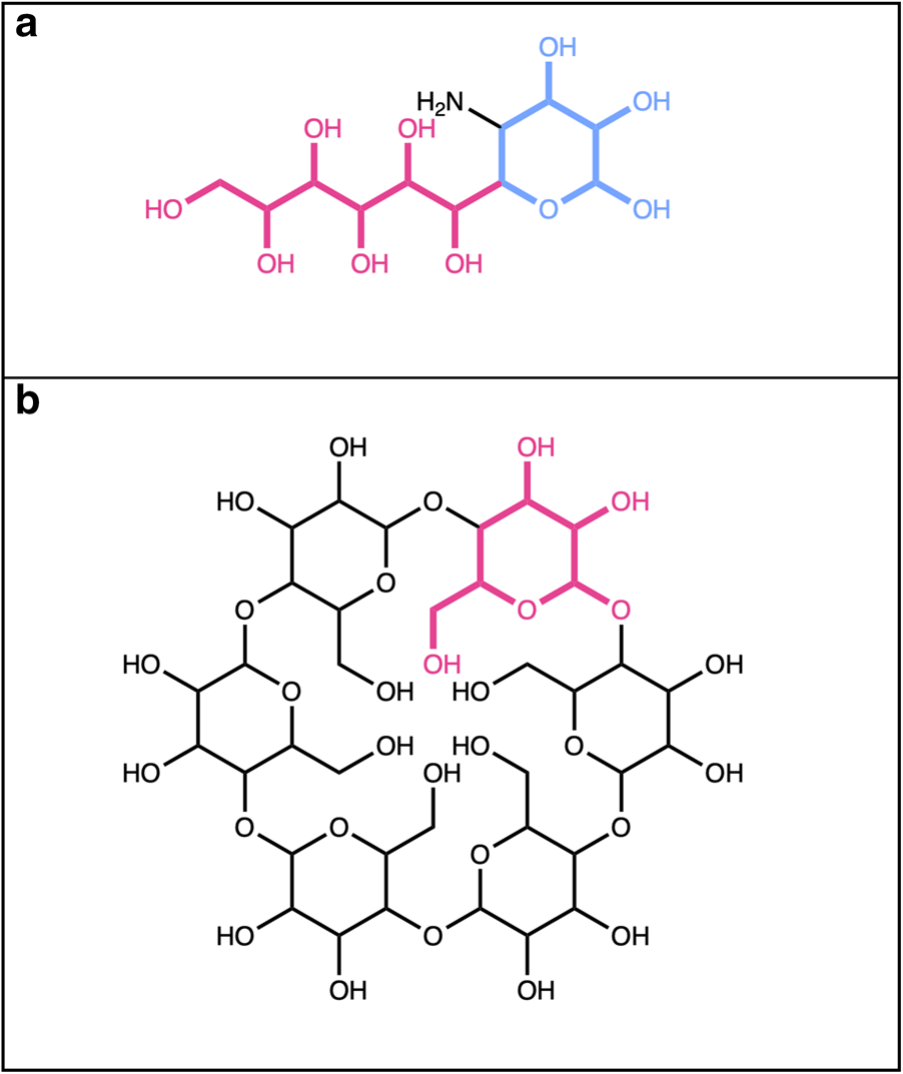
**a** 1-(3-Amino-4,5,6-trihydroxyoxan-2-yl)hexane-1,2,3,4,5,6-hexol is composed only of one circular sugar (in blue) with an exocyclic nitrogen and a linear sugar alcohol (in red). **b** Cyclodextrin, a macrolide composed only of circular sugars linked by glycosidic bonds; in red, one of the circular sugar units

Figures 12, 20a both show molecules that are only composed of different, circular and linear sugar moieties: both molecules get entirely removed if all, or only terminal, linear and circular sugars are to be detected and removed. It is however not the case for cyclodextrin (Fig. 20b), a macrolide composed only of circular sugars. The big cycle is detected as a macrocycle by the SRU and therefore all the small circular sugars share bonds with it, which makes them fused instead of isolated and therefore undetectable by the circular sugar detection algorithm.

### Comparison with pre-existing algorithms

The validation of the SRU cannot be done otherwise than visually and with a known reference dataset, as there is not, for now, a reference algorithm that the performance can be compared to. CTPIC [16], published recently, is the only work that tackles the sugar detection problem in biological molecules. However, this probabilistic approach is designed to distinguish if a molecule is a sugar or is derived from one, from a molecule that is not. It does not detect solely sugar moieties in the molecular substructures, nor removes them, and therefore cannot directly be compared to the SRU.

## Discussion

Chemical and enzymatic deglycosylation of molecules is a widely studied and relatively well-known process and has various applications in both research and industry. On the other hand, theoretical deglycosylation of molecules, and in particular of NPs, is also frequently mentioned in cheminformatics approaches to study molecular structure and properties without the influence of redundant and monotonous sub-structures such as sugars. However, despite the general acknowledgement of its importance, no commonly agreed algorithm and open-source implementation of such deglycosylation has been made available. In some studies, it is just noted that sugars were removed with an in-house algorithm, in some others, it is mentioned which sugars have been removed, but not exactly how. Scaffold Hunter is the only open software, that the authors are aware of, which contains a method for terminal ring sugar removal. In this context, the necessity of an exhaustive and open-source approach for an in silico sugar removal algorithm is therefore obvious. The present work is an attempt to fill this gap, with a systematic in silico deglycosylation algorithm, its code, a standalone command-line application and a web application.

### Specificities of sugar detection

Four main sugar moiety types are defined here: terminal ring sugar, non-terminal ring sugar, terminal linear sugar and non-terminal linear sugar (Fig. 1). The difference between a ring and a linear sugar is trivial, but the notion of a terminal and non-terminal substructure had to be clearly defined, in order to be able to target only substructures of interest. A molecular substructure is said to be terminal when its removal does not split the parental structure into two or more disconnected structures. Opposed to this, a molecular substructure is said to be non-terminal when its removal results in two or more disconnected structures. However, this definition leads to cases where a sugar moiety is detected as non-terminal but would be perceived as terminal by a human expert. This happens in cases where a terminal sugar moiety carries an additional function as a methyl, amine, or phosphate group (Figs. 14a, c, 15f, 17c, 20a). The SRU perceives as being the circular sugar moiety only the central tetrahydrofuran, tetrahydropyran, and oxepane rings (Fig. 5), therefore additional substructures, like hydroxy groups, that are naturally parts of sugar moieties (Fig. 8b) would get disconnected from the parent structure and the sugar ring will be detected as non-terminal. To avoid this, a non-optional parameter has been introduced to the SRU, the “preservation mode”. This mode evaluates the size of each disconnected substructure after the removal of a detected sugar moiety, and if its size is under the predefined threshold, it will be removed together with the sugar. The default threshold to keep the substructures is 5 heavy atoms, which guarantees a safe removal of the hydroxy and methyl groups, but preserves more interesting substructures. After this step, if more than one unconnected substructure remains, i.e. not just the deglycosylated parent structure, the sugar moiety is classified as non-terminal. Of course, it is also possible to preserve all substructures by setting the preservation mode accordingly.

Initially, the detections of circular and linear sugar moieties were separated because the CDK ring detection functionalities allows a straightforward extraction of substructures relevant for circular sugar detection, i.e. isolated cycles. For linear sugars, on the other hand, a substructure match over the whole molecule has to be performed. The substructures used for this initial detection (Fig. 6) were compiled using structures of commonly known sugars and insights about linear sugar moieties appearing in NPs. Since overlapping matches of these patterns in the target molecules are combined in the following step, the chosen motifs can also be considered as “building blocks” of the diverse forms of linear sugars encountered in NPs. The focus on NPs resulted in the inclusion of acidic sugars in the pattern set (Fig. 6), as they appear in NPs and can be considered sugar-like. Nevertheless, the SRU makes it optional to detect them because they do not adhere to a strict definition of sugars. Due to the step where combining overlapping substructure matches, very small structures, like triols, and also very big structures can become linear sugar candidates. The latter are shortened in most cases by the splitting of ether, ester and peroxide bonds. These series of splitting and combining candidate substructures can lead to candidates of various sizes, therefore we defined the minimal and the maximal size of a single sugar moiety within this framework. These minimum and maximum size parameters of linear sugars are defined as carbon atom counts, by default 4 and 7 carbons, and can be adjusted in the SRU.

The hydroxy groups are not explicitly included in the sugar ring detection by the SRU, however, their presence makes the important difference between, for example, a bare tetrahydropyran ring (Fig. 5) and a glucopyranose moiety (Fig. 8), with all possible intermediate forms. To specify which hydroxylation level should be classified as a sugar, a threshold, specifying the ratio of exocyclic oxygen atoms divided by the number of atoms in the ring, was introduced in the circular sugar detection algorithm. Its default value is set to 0.5: with this a pyranose ring needs at least 3 connected exocyclic oxygen atoms to be classified as a sugar ring. For cases where the hydroxylation of the detected sugars is of minor importance, this option can be switched off.

Functional groups like keto groups can be encountered in NP structures that, at first glance, could be perceived as circular sugars. In the development of the SRU it was decided to exclude such structures, together with all those that have exocyclic double or triple bonds from being recognized as sugars, as they do not comply with the traditional definition of sugars. Additionally, molecular features like a hydroxy group that got oxidized to a keto group in biosynthesis of an NP are considered too significant to get removed.

The next important point to discuss when talking about sugar moieties, is how they are connected between them or to the parent structure. In many, but not all cases, ring sugars are connected to the above-mentioned structures by an O-, N-, S- or C-glycosidic bond. The SRU, by default, allows removing all detected ring sugars regardless of their connection to the core structure of the aglycon or, as an option, only those connected by an O-glycosidic bond. The three other glycosidic bond types (N-, S- and C-) are rare and might be considered as too interesting to be removed. It is to be noted that the O-glycosidic bond that the SRU detects is defined as an oxygen atom connected to one carbon of the circular sugar moiety in any position and to another non-hydrogen atom outside of the sugar ring. This definition includes therefore linkages like ester bonds, when the sugar moiety is connected to a single-bonded oxygen atom. However, when the submitted molecule is composed only of a circular sugar, it will be removed in all cases, even if the option to detect the glycosidic bond was set, as there is no other structure within such a molecule to bond to.

The last important aspect raised during the SRU development and testing, was the detection of linear sugars within molecules containing macrocycles. Macrocycles are molecules that contain more than ten membered rings and when their backbone is made of carbon atoms, with few oxygens and no double or triple bonds, they easily match with linear sugar patterns, in particular sugar alcohols and ketoses. In most of cases, the macrocycle needs to be preserved, therefore the option “detect linear sugars in rings” was introduced. This option detects the linear sugars within a cycle but will discard the hit to prevent its removal if turned off.

### SRU efficiency

A previously published manually curated set of sugars encountered in bacterial NPs [6] has been used to validate the consistency of the sugar detection and removal of the SRU. It detected as being a sugar more than the half (58%) of the moieties in this set, which might seem as poor performance. However, the remaining 42%, after manual verification, do not correspond to the canonical definitions of sugar moieties, due to the presence, for example, of a double bond or of a non-oxygen atom in the sugar cycle. The SRU then has been proven to efficiently detect canonical circular and linear sugars that are encountered in bacteria. Then, a small manual dataset of complex natural products with linear and circular and terminal and non-terminal sugar moieties in their structures (Figs. 13, 14, 15, 16, 17, 18, and 19) has been assembled to observe the SRU behavior in non-trivial cases. All the described options and special cases have been successfully tested. In general, the SRU algorithm shows a good balance between removing repetitive, uninteresting structures and keeping the interesting ones, especially considering the variety of sugar molecules present in natural products as illustrated in both the review data and the manually assembled set. Adapting the various settings according to one’s specific needs can also make a high impact on the results of the sugar detection and removal.

We were able to process the COCONUT dataset [21] with 427,000 molecules in 11 min and a ZINC [22] subset with 3 mio molecules in 21 min on an up-to-date laptop using the SRU command-line application, which includes the time for reading the molecules from SDF. The comparatively shorter time required for the larger dataset can be attributed to the less frequent occurrence of sugars in the mainly synthetic molecules from ZINC. Dividing the dataset into chunks and processing them in parallel on a multi-core machine will likely lead to near-linear speed-up with the number of employed cores.

### Future developments

The deglycosylation algorithm presented in this manuscript contains several non-crucial limitations, and can be subject to improvement. For instance, the stereochemistry of the sugars to be removed and of the query molecules is not taken into account.

The Daylight SMARTS (SMILES arbitrary target specification) patterns [23] describe substructural patterns in molecules and are based on the SMILES molecular representation. The rising interest of the scientific community in SMARTS patterns, because of their practical and easy application makes it tempting to integrate them in any substructure detection application. However, building a SMARTS pattern is not easy, and is particularly challenging for sugars due to their versatility, repeated units and numerous exceptions. One future step in the development of the SRU will be to develop and integrate general SMARTS patterns for both ring and linear sugars that also take into account their connectivity particularities.

The sugar removal algorithm should also be added to the official CDK repository to avoid users downloading separate libraries and to easily implement the sugar removal processes in their own software and workflows. The substructure removal algorithm can also be extended to allow removal of lipids and amino acids, as they can also be highly repetitive and redundant in NPs.

## Conclusion

We presented an algorithm for standardized sugar removal from natural products and from molecular structures in general. The standalone SRU command-line application offers maximal flexibility regarding the way the sugar moieties can be detected and removed. The most prominent choice to make is to whether remove only linear or circular sugar moieties or both from the submitted molecules. The other options include settings already discussed, like the removal of all detected moieties or only the terminal ones, whether linear sugars in rings should be detected and removed, the preservation mode option to use and its threshold, whether possible circular sugars must have a glycosidic bond or a sufficient number of exocyclic oxygen atoms attached (with threshold), whether sugar-like spiro rings and linear acidic sugars should be included, and the minimum and maximum sizes for linear sugars. Even more options are available when the SRU is used as a library in other software. This way, for instance, the circular sugar patterns used can be easily altered to, for example, remove only 5- and 6-membered rings or to also include the 8-membered rings to be removed. Also, the linear sugar patterns used for the initial detection of linear sugar candidates can be configured. The SRU source code can also be used to only detect sugar moieties in given molecules without removing them or to return the removed moieties along with the aglycon after sugar removal.

We hope that this will lead to a better reproducibility in cheminformatics analyses requiring the removal of such abundant substructures as sugar moieties. The Java code for the CDK-based implementation is available on GitHub (https://github.com/JonasSchaub/SugarRemoval). A user-friendly web application for sugar removal is available at https://sugar.naturalproducts.net/.

## Supplementary information

**Additional file 1.** For the sugar moieties structures that have been used in the tests.

## Data Availability

Data and software are freely available under the MIT license. The source code for the sugar removal algorithm is available on GitHub at https://github.com/JonasSchaub/SugarRemoval, along with the standalone command-line application downloadable as a JAR. The web application is available at https://sugar.naturalproducts.net/ and its code is available at https://github.com/mSorok/SugarRemovalWeb.

## Abbreviations

SRU: Sugar Removal Utility
NP: Natural product
CDK: Chemistry Development Kit
